# Emerging Role of GLP-1 Agonists in Obesity: A Comprehensive Review of Randomised Controlled Trials

**DOI:** 10.3390/ijms241310449

**Published:** 2023-06-21

**Authors:** Mihaela-Simona Popoviciu, Lorena Păduraru, Galal Yahya, Kamel Metwally, Simona Cavalu

**Affiliations:** 1 Faculty of Medicine and Pharmacy, University of Oradea, P-ta 1 Decembrie 10, 410073 Oradea, Romania; 2 Department of Microbiology and Immunology, Faculty of Pharmacy, Zagazig University, Al Sharqia 44519, Egypt; 3 Department of Molecular Genetics, Faculty of Biology, Technical University of Kaiserslautern, Paul-Ehrlich Str. 24, 67663 Kaiserslautern, Germany; 4 Department of Medicinal Chemistry, Faculty of Pharmacy, University of Tabuk, Tabuk 71491, Saudi Arabia; 5 Department of Pharmaceutical Medicinal Chemistry, Faculty of Pharmacy, Zagazig University, Zagazig 44519, Egypt

**Keywords:** obesity, GLP-1 agonists, weight loss, liraglutide, semaglutide, tirzepatide, Lixisenatide, exenatide, type 2 diabetes

## Abstract

Obesity is a chronic disease with high prevalence and associated comorbidities, making it a growing global concern. These comorbidities include type 2 diabetes, hypertension, ventilatory dysfunction, arthrosis, venous and lymphatic circulation diseases, depression, and others, which have a negative impact on health and increase morbidity and mortality. GLP-1 agonists, used to treat type 2 diabetes, have been shown to be effective in promoting weight loss in preclinical and clinical studies. This review summarizes numerous studies conducted on the main drugs in the GLP-1 agonists class, outlining the maximum achievable weight loss. Our aim is to emphasize the active role and main outcomes of GLP-1 agonists in promoting weight loss, as well as in improving hyperglycemia, insulin sensitivity, blood pressure, cardio–metabolic, and renal protection. We highlight the pleiotropic effects of these medications, along with their indications, contraindications, and precautions for both diabetic and non-diabetic patients, based on long-term follow-up studies.

## 1. Introduction

### 1.1. Prevalence of Obesity Globally

Obesity is a chronic disease that has become a major concern in recent years, as it can have negative impacts on health, leading to increased morbidity and mortality. A body mass index (BMI) is the most commonly used measurement for assessing the prevalence of obesity. The World Health Organization (WHO) defines BMI as a simple weight-for-height index used to classify underweight, normal weight, overweight, and obesity in adults. BMI is calculated as weight in kilograms divided by the square of height in meters (kg/m^2^) [1].

Adults with a BMI of 25.0 to 29.9 kg/m^2^ are considered overweight, while those with a BMI of 30 kg/m^2^ or higher are considered obese. For children and teenagers aged 2 to 18, the BMI scale is not utilized, and instead, a percentile scale based on gender and age is advised. In this demographic, obesity is defined as a BMI at or above the 95th percentile, while overweight is defined as a BMI in the 85th to 94th percentile. Increasing BMI has been associated with increased mortality rates, with a 29% increase in overall mortality, a 41% increase in vascular mortality, and a 210% increase in diabetes-related mortality for every 5-unit increase in BMI over 25 kg/m^2^. Central adiposity, as indicated by increased waist circumference [2] and other measures, can predict the cardiometabolic risk that cannot be identified by BMI alone. For instance, the abdominal circumference can be correlated with BMI such that in women of normal weight (BMI 18.5–24.9 kg/m^2^), the circumference is ≥80 cm, and in men, it is ≥90 cm [3]. In May 2022, the World Health Organization published a report that revealed almost 60% of European adults are overweight or obese, and in children under the age of five, this percentage is 7.9% or approximately 4.4 million in Europe. Although the prevalence decreases temporarily in those aged 10–19 years, there have been consistent increases in the prevalence of overweight and obesity in the WHO European Region, and no Member State is on track to reach the target of halting the rise in obesity by 2025.

The report predicts that in the coming decades, obesity will overtake smoking as the leading preventable risk factor for cancer in some countries. It also emphasizes that obesity is a condition that needs to be specifically treated and managed, not just a risk factor. The European Region has the second-highest prevalence of adult obesity among all WHO regions, after the Americas Region. Overweight and obesity cause more than 1.3 million deaths globally each year, with even these figures possibly underestimated.

In the European Region, overweight and obesity have reached epidemic proportions, with higher prevalence levels among men (63%) than women (54%). Rates tend to be higher in higher-income countries, and the highest levels of overweight and obesity are found in Mediterranean and Eastern European countries. Educational inequalities are widespread, with a higher prevalence of obesity in people with lower educational levels [4].

**Figure 1:**
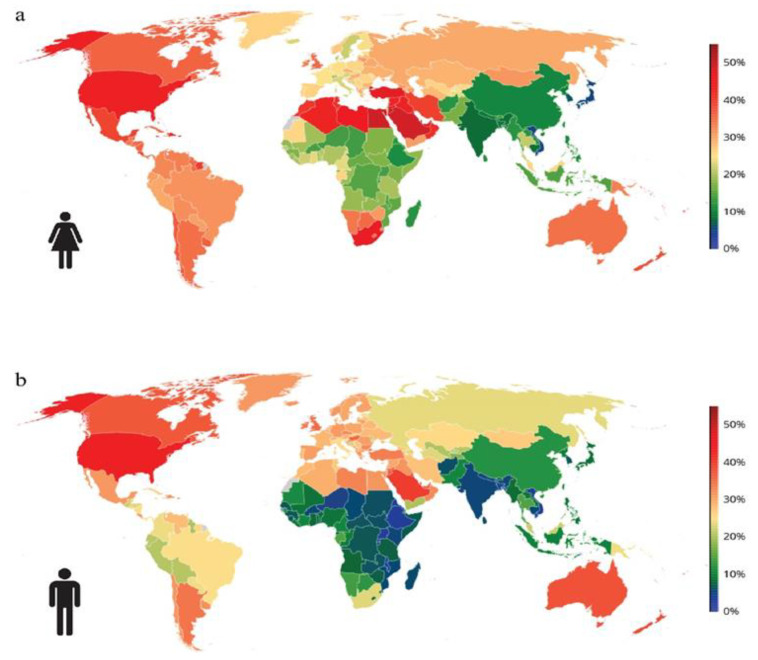
The worldwide prevalence of obesity in 2025: (**a**) females and (**b**) males. Obesity refers to BMI ≥ 30 kg/m^2^. Age-standardized estimates for adults aged 20 years and older. Data obtained from NCD-RisC study. Available online: https://www.ncdrisc.org (accessed on 15 February 2023).

A study conducted on the Romanian population confirmed the previously mentioned figures regarding the increase in the frequency of weight and obesity. The Obesity in Romania (ORO) study included a sample of 2103 participants with an average age of 41.5 years and an average BMI of 29.9 kg/m^2^ (range: 15.2 to 57.8 kg/m^2^). The estimated prevalence of overweight was 31.1%, and obesity was 21.3%, with the highest prevalence being in the age range of 60–79 years, at 41.6% [5,6]. The worldwide prevalence of obesity is represented in Figure 1.

### 1.2. Complications of Obesity

**Figure 2:**
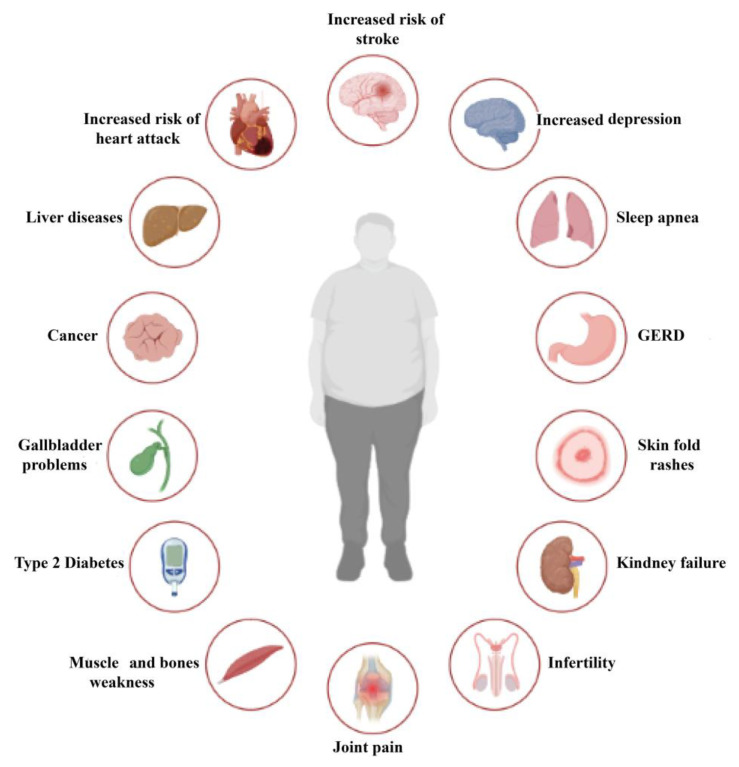
Complications of obesity.

Obesity is a multifaceted and diverse condition that affects more than just an individual’s weight [7]. Although body mass index (BMI) is a widely used tool to diagnose obesity, it fails to capture the full extent of the health consequences associated with excess weight [8]. Obesity-related complications are comparable to those of other chronic diseases, resulting in higher rates of morbidity and mortality [9,10]. Biomechanical complications, including osteoarthritis and obstructive sleep apnea, arise from excess adipose tissue, while adipose tissue dysfunction contributes to cardiometabolic complications. Cardiometabolic disease starts with insulin resistance, which initially goes unnoticed but can progress to metabolic syndrome, prediabetes, elevated blood pressure, dyslipidemia, and hepatic steatosis. These conditions indicate the risk of developing terminal complications such as type 2 diabetes mellitus (T2DM), nonalcoholic steatohepatitis, and cardiovascular disease [11]. Obesity promotes insulin resistance development and leads to the progression of cardiometabolic disease towards these severe consequences [12]. The main complications and effects of obesity can be summarized in Figure 2.

The literature widely supports the notion that obesity is linked to various health complications [1]. Common cardiovascular complications include hypertension [13], atherosclerosis, heart failure [14], and atrial fibrillation [15], while metabolic complications include T2DM [16,17], dyslipidemias [18], hyperuricemia, and metabolic syndrome [19]. Respiratory complications such as mixed ventilatory dysfunction, Pickwick syndrome, sleep apnea syndrome [20], asthma [21], and digestive complications such as gastroesophageal reflux [22,23], hiatal hernia, gallstones, and non-alcoholic fatty liver [24] are also often observed in obese individuals.

Additionally, obesity is associated with osteoarticular complications such as arthrosis [9], venous and lymphatic circulatory disorders [25], endocrine complications including hyperinsulinism, hypercorticism, hypothyroidism, and hypogonadism [26,27], and oncological conditions such as endometrial [28,29] and breast cancer [30], esophageal, liver cancer, colorectal, prostatic, thyroid, pancreatic adenocarcinoma, renal, and multiple myeloma [31]. Genitourinary complications such as menstrual disorders, reduced fertility, obstetric complications, polycystic ovary [32,33], gynecomastia, urinary incontinence, and impaired renal function [34] have also been associated with obesity (Figure 2).

Cutaneous complications like bacterial and fungal infections [35] and mental disorders such as depression, binge eating disorder, and anxiety disorders [36,37] have also been reported in obese individuals. Moreover, obesity is linked to an increased risk of hospitalization, the need for intubation and ventilation, and the risk of death in cases of COVID-19 and 2009 H1N1 [38].

It is important to note that the severity of complications associated with obesity can vary depending on individual factors such as overall health, lifestyle, and other medical conditions.

### 1.3. The Emergence of Glucagon-Like Peptide 1 Receptor Agonists and the First Results Obtained upon Administration of Exenatide

Historically, short-term weight loss medications such as phentermine, benzphetamine, and diethylpropion were approved for use over several weeks in the 1960s [39]. However, long-term safety data for these medications are not available [40]. Orlistat, which acts intraluminally to influence intestinal fat digestion and absorption, was approved for chronic weight management in 1999 [41]. Recent research has shown that improper interactions between satiety hormones and the central nervous system’s feeding centers are responsible for excessive adipose tissue mass (CNS). Specifically, interactions between orexigenic hormones such as ghrelin and anorexigenic hormones like leptin, cholecystokinin, peptide YY (PYY), and amylin with the hypothalamic satiety centers lead to a caloric intake level that produces and maintains excess adiposity. Maladaptive reactions that occur after weight loss are also significant components of the pathophysiology of obesity. A hypocaloric diet that causes weight loss leads to a decrease in anorexigenic hormones such as glucagon-like peptide-1 (GLP-1), amylin, cholecystokinin, and PYY while increasing orexigenic hormones such as ghrelin [42]. To continuously address these issues, drugs that can suppress appetite by correcting defects in the satiety hormone CNS axis are required. Fenfluramine, sibutramine, and lorcaserin were three such FDA-approved medications discontinued due to safety concerns. However, between 2012 and 2014, three centrally acting medications—extended-release phentermine/topiramate (ER), an opioid receptor antagonist combined with a dopamine/norepinephrine reuptake inhibitor used for depression, liraglutide 3 mg/day, and naltrexone ER/bupropion (ER)—were approved for chronic weight management. These medications are still available to clinicians as GLP-1 receptor agonists (GLP1-RAs) [10]. All of them met FDA efficacy standards in randomized phase 3 clinical studies, with patients in the placebo group losing an average of 5% of body weight, or 35% more than the control group. In June 2021, semaglutide 2.4 mg administered subcutaneously once a week became another FDA-approved (GLP1-RA) for chronic weight management [43]. Compared to the data for previously approved obesity medications, the phase 3 randomized controlled trial data for this treatment showed nearly twice the weight loss. The approval of medication with this level of efficacy will open up a “new vista” for the treatment of obese individuals [12]. The FDA’s approval of oral semaglutide, the first oral GLP-1 RA, signals a paradigm shift in treating T2DM. Patients with T2D who require better glycemic control, want to lose weight, and are not interested in injectable medication may find oral semaglutide to be an appealing alternative [44].

The therapeutic management of people with Type 2 DM requires a holistic approach centered on the patient and should be as free as possible of adverse effects and with a strong impact in the reno–cardio–metabolic protection, sustainable, durable, and robust, to cover the extremely complex pathogenic mechanisms. Nowadays, it is accepted that in addition to the main benefit in metabolic control, GLP1-RA show benefits in weight loss for both people with diabetes and those without, a decrease maintained over time without Yo-Yo phenomena. These aspects bring us to perspective on the possibility of prescribing these molecules in the treatment of people with obesity with or without diabetes. However, we strongly need evidence from the real-life experience of the therapeutic benefits under conditions of maximum cardiovascular and renal safety and anti-atherosclerotic protection. In this context, the aim of this review is to highlight the active role of GLP-1 agonists and the main outcomes demonstrated by clinical trials in diabetes and non-diabetic obesity aiming to reduce morbidity and mortality associated with these metabolic disorders and to improve the quality of life.

## 2. Design of GLP1-RA

**Figure 3:**
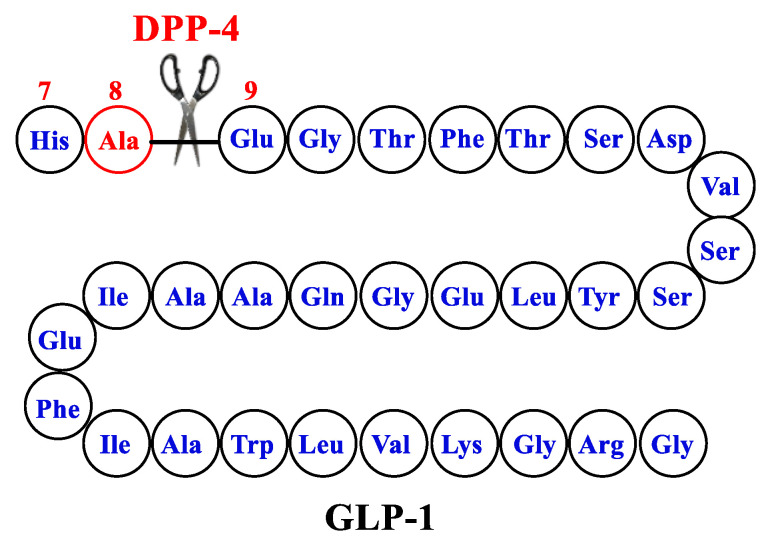
The structure of GLP-1-parent.

GLP-1 by itself is not an ideal therapeutic agent because of its very short half-life (about 2 min), which is attributed to rapid degradation by dipeptidyl peptidase 4 (DPP-4). In addition, GLP-1 is rapidly cleared by the kidneys due to high hydrophilicity. DPP-4 metabolizes GLP-1 by cleaving the peptide chain between Ala-8 and Glu-9 (Figure 3). Two approaches have been adopted to improve the pharmacokinetic profile of GLP-1. The first approach aimed at prolonging the half-life of GLP-1 by inhibiting its degradation by DPP-4 through the development of DPP-4 inhibitors. This approach furnished several clinically useful orally bioavailable small-molecule drugs in the drug market, such as sitagliptin, saxagliptin, linagliptin, and vildagliptin. The other alternative approach involved the development of GLP-1 RAs resistant to degradation by DPP-4. Here, we review the design of different GLP1-RAs starting from their parent GLP-1.

**Figure 4:**
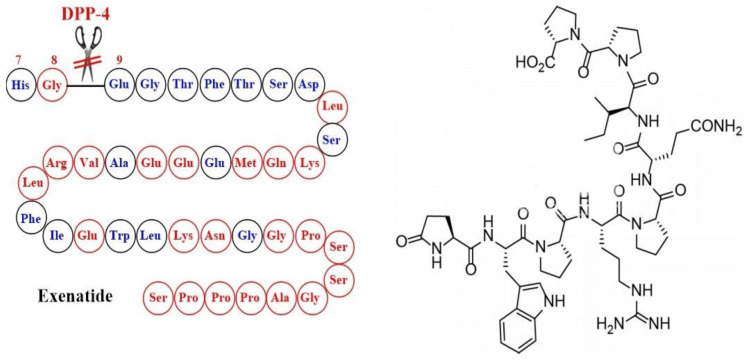
Development of exenatide and the corresponding chemical structure.

The first GLP1-RA with useful therapeutic activity was inspired by nature through the identification of exendin-4, which is a natural peptide hormone isolated from the saliva of the venomous lizard Gila monster (*Heloderma suspectum*). Exendin-4 was found to display similar activity to GLP-1 but with a markedly longer half-life (2–4 h) because of its enhanced resistance to DPP-4 attributed to the replacement of Ala-8 by Gly-8 at the cleavage site [45] (Figure 4). The synthetic version of exendin-4 (exenatide) was approved by FDA in 2005 in the parenteral form to improve glycemic control in adults with T2DM as an adjunct to diet and exercise [46]. The shortcomings of exenatide therapy include unsatisfactory circulation half-life due to rapid renal clearance and potential immunogenicity.

**Figure 5:**
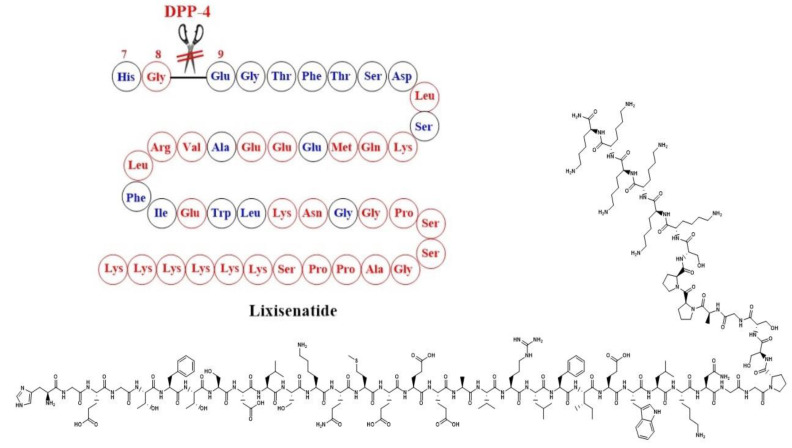
Development of Lixisenatide and the corresponding chemical structure.

Lixisenatide is a structural analogue of exendin-4 where six lysine residues were added at the C-terminus with the deletion of a proline residue [47] (Figure 5).

One of the successful strategies to inhibit both renal clearance and DPP-4 binding involves the conjugation of GLP1-RA with hydrophobic long-chain fatty acid moieties capable of binding non-covalently to albumin. These conjugates exceed the size limit for glomerular filtration, and consequently, their renal clearance is diminished, and their circulation half-life is increased. Three long-acting drugs in the market represent this approach which are liraglutide, semaglutide, and tirzepatide.

**Figure 6:**
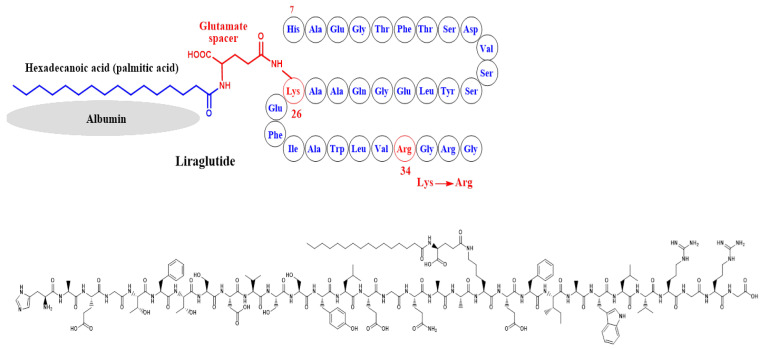
Development of liraglutide and the corresponding chemical structure.

In liraglutide, two modifications were performed on the structure of GLP-1, which are the attachment of a C-16 palmitoyl acid moiety to Lys-26 via a glutamate spacer and the replacement of Lys-34 by Arg-34 [48] (Figure 6). These structural modifications extended the circulation half-life to about 13 h.

**Figure 7:**
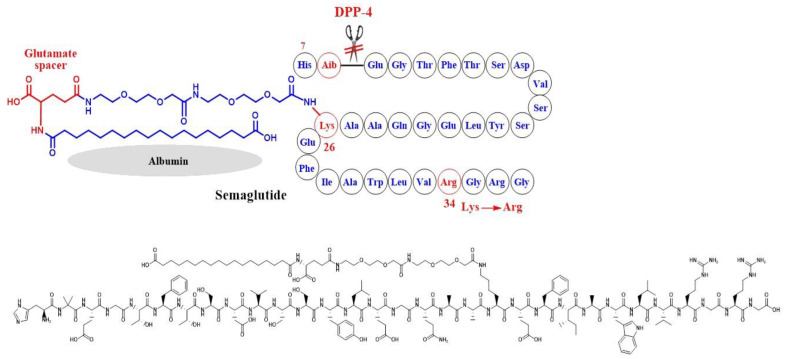
Development of semaglutide and the corresponding chemical structure.

In semaglutide, Lys-26 was attached to a hydrophobic C-18 fatty di-acid moiety to bind to albumin and inhibit glomerular filtration due to its high molecular size (Figure 7). In addition, Ala-8 at the cleavage site of GLP-1 was replaced by an unnatural amino acid which is 2-aminoisobutyric acid (AIB), aiming to inhibit degradation by DPP-4 [49]. In both examples, Lys-34 was replaced by Arg-34, probably to optimally orient the fatty acid moiety for albumin binding (Figure 7).

**Figure 8:**
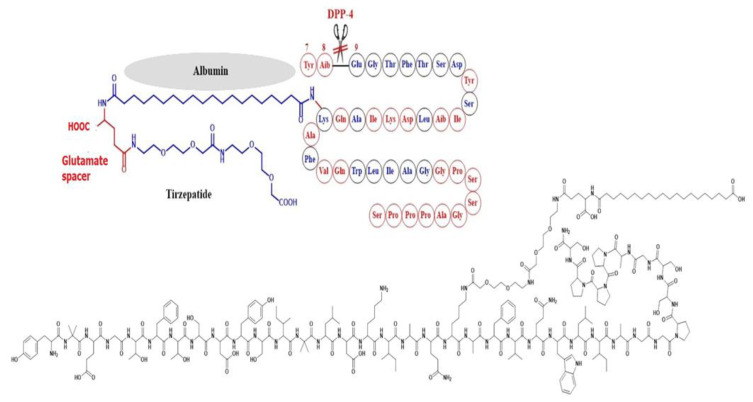
Development of tirzepatide and the corresponding chemical structure.

Tirzepatide was developed by Eli Lilly in 2022 and considered by FDA as a first-in-class drug as it is dually acting as a GLP-1 RA and GIP-RA (glucose-dependent insulinotropic peptide receptor agonist). The molecule combines some of the structural features of both GLP-1 and exenatide. Ala-8 was replaced by the unnatural amino acid 2-aminoisobutyric acid (Aib) to inhibit degradation by DPP-4, and Lys-20 was attached to a C20 fatty diacid moiety through a spacer in order to bind albumin and hence diminish renal clearance [50] (Figure 8).

**Figure 9:**
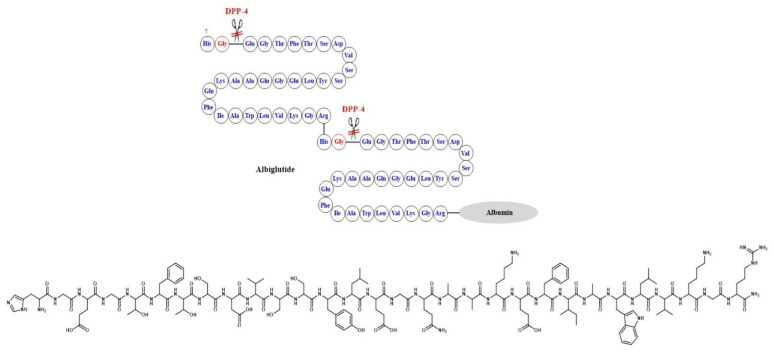
Development of albiglutide and the corresponding chemical structure.

In albiglutide, however, a different strategy was used where two copies of DPP-4 resistant GLP-1 analogues were linked through the N-terminus, and the terminal arginine was attached to albumin. The large molecular size of the albiglutide–albumin hybrids inhibits renal clearance and hence prolongs its circulation half-life. DPP-4 degradation was diminished through a Gly8Ala mutation at the cleavage site of the GLP-1 molecule [51] (Figure 9).

## 3. Involvement of the Incretin System in Obesity

**Figure 10:**
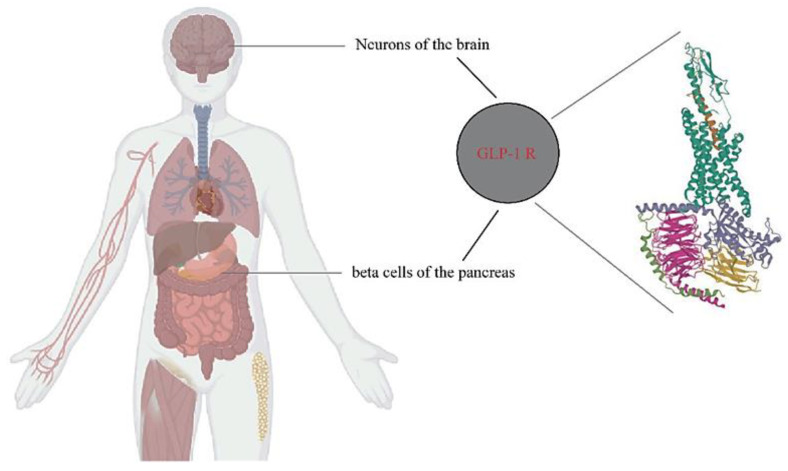
Distribution of GLP-1 receptors. GLP-1Rs (pdb6X18) mainly exist in the neurons of the brain and the beta cells of the pancreas.

Various signals from the peripheral organs send information to the central nervous system about the nutritional status [52,53]. Despite the exploration of the potential therapeutic utility of these peripheral signals, including the use of incretins, a group of metabolic hormones that lower blood sugar levels by increasing insulin secretion from the pancreas, inhibiting the secretion of glucagon, and reducing nutrient absorption, no successful treatments for obesity have been found [54,55]. Incretins consist of GLP-1 and GIP, which belong to the glucagon peptide superfamily and are rapidly inactivated by DPP-4 [56]. GLP-1 has several effects on various organ systems, among which the most relevant is the reduction of appetite and food intake, leading to long-term weight loss. GLP-1 secretion from the gut seems to be impaired in obese subjects, suggesting a role in the pathophysiology of obesity [57]. GLP-1 RAs are currently used in treating patients with T2D and consistently result in weight loss, in addition to lowering blood glucose levels. The combined central and peripheral actions of GLP-1 RA promote satiety, decrease hunger, and ultimately reduce food intake. While GLP-1 RA-induced deceleration of gastric emptying and occasional nausea may contribute to the weight-reducing effects, they appear to play a minor and temporary role [58]. The inhibition of food intake by GLP-1/RA-mediated GLP-1 has been attributed to both direct central actions, with GLP-1 receptors present in brain regions involved in food intake and energy balance, and indirect pathways via vagal afferents originating in the gut and portal circulation [59,60] (Figure 10). In rodents, intra-cerebrovascular injection of GLP-1 reduced food intake. However, in addition to the direct effects of GLP-1 on the CNS, the incretin more likely exerts its actions on the brain through indirect pathways, that is, through vagal afferents originating in the gut and portal circulation [59,60].

Exenatide was the first GLP-1 RA to be approved for the treatment of T2DM in 2005 [61]. Since then, GLP-1 RAs have undergone further development, using certain compounds/preparations that have resolved the initial issue of rapid elimination (resulting in a short half-life), which necessitated frequent injections (such as twice a day for bid exenatide) [61].

Currently, there are GLP-1 RAs that are injected once daily (Lixisenatide and liraglutide), twice daily (exenatide bid), or once weekly (dulaglutide, albiglutide, and semaglutide). Recently, a daily oral semaglutide formulation that showed clinical efficacy comparable to the once-weekly subcutaneous preparation was approved [61,62]. Common mechanisms of action for all GLP-1 RAs include increased insulin secretion induced by hyperglycemia, suppression of glucagon secretion in conditions of hyper- or euglycemia, sluggish gastric emptying that prevents significant increases in postprandial blood glucose, and a decrease in caloric intake and implicit body weight.

Although short-acting medications (such as exenatide bid and Lixisenatide) are less successful at lowering blood sugar levels throughout the night and in the morning, they continue to have a positive impact on gastric emptying when used in conjunction with basal insulin and/or long-term therapy [63]. Since 2016, numerous cardiovascular (CV) trials have demonstrated that GLP-1 RAs are a useful means of reducing mortality due to CV events such as acute myocardial infarction and stroke. As a result, recommendations prioritize GLP-1 RA treatment for individuals with atherosclerotic vascular disease (such as previous CV events) [64]. The individual risk of ischemia or heart failure consequences should guide the treatment choice because sodium/glucose cotransporter-2 (SGLT-2) inhibitor therapy also lowers CV events (with impact predominantly driven by a reduction in heart failure complications). GLP-1 RAs might aid in preventing type 2 diabetes’ renal consequences [65].

## 4. Liraglutide

Liraglutide was the first daily injectable GLP-1 RA approved for use in people with T2DM. The LEAD studies compared liraglutide with other drugs used for diabetes.

### 4.1. Evidence from Clinical Trials in Diabetics and Non-Diabetics

The LEAD series, which commenced in 2006 and involved 1041 adults from 21 countries, investigated the efficacy and safety of liraglutide, a human GLP-1 analog, in the treatment of type 2 diabetes mellitus (T2DM). The initial study in the series demonstrated that once-daily liraglutide when added to sulfonylurea therapy for 26 weeks, resulted in greater improvements in glycemic control and weight loss compared to the addition of rosiglitazone or placebo [66]. In the LEAD-2 trial, the effectiveness of adding metformin to T2DM patients who had previously received oral antidiabetic medication was assessed by adding a placebo or glimepiride. When both medications were given metformin as a background treatment, it was found that once-daily liraglutide, compared to glimepiride, provided comparable glycemic control, weight loss, and reduced the occurrence of hypoglycemia [67]. The LEAD-3 study investigated the safety and efficacy of liraglutide as monotherapy in 746 patients with early-stage T2DM. The results indicated that liraglutide is a safe and effective initial pharmacological therapy for T2DM, resulting in greater reductions in glycosylated hemoglobin, weight, hypoglycemia, and blood pressure compared to glimepiride [68]. In the LEAD-4 study, liraglutide was administered in combination with metformin and thiazolidinedione in 533 T2DM patients for 26 weeks. The study revealed that liraglutide, metformin, and thiazolidinedione constituted a well-tolerated combination therapy for T2DM that significantly improved glycemic control [69].

The LEAD-5 study examined liraglutide as an alternative treatment for T2DM patients who were candidates for insulin glargine. The study involved 230 patients treated with liraglutide, 114 treated with a placebo, and 232 treated with insulin glargine. liraglutide was found to significantly reduce glycosylated hemoglobin compared to glargine and placebo and provided better glycemic control and weight outcomes [70]. In the final study of the LEAD series, LEAD-6, liraglutide was compared to exenatide twice daily in a 26-week treatment of T2DM patients. The results showed that liraglutide provided significantly better glycemic control than exenatide twice daily and was better tolerated [70]. These findings suggest that liraglutide may be a viable treatment option for T2DM, particularly when weight loss and the risk of hypoglycemia are major concerns [70].

### 4.2. Liraglutide at A Dose of 3 mg/Day in Non-Diabetics

The prevalence of obesity has dramatically increased in recent years, and few safe and effective drugs are currently available to treat it. This has prompted studies evaluating the effect of liraglutide on body weight and tolerability in obese people without T2DM. One such study randomly assigned 564 individuals (18–65 years old, BMI 30–40 kg/m^2^) to one of four doses of liraglutide (1.2 mg, 1.8 mg, 2.4 mg, or 3.0 mg, *n* = 90–95) or placebo (*n* = 98) administered subcutaneously once daily, or to orlistat (120 mg, *n* = 95) administered orally three times daily. All subjects were on an energy-deficient diet of 500 kcal per day and increased their physical activity throughout the study, including a two-week period. Those who received liraglutide lost significantly more weight than those who received a placebo or orlistat. Mean weight loss with liraglutide 1.2–3.0 mg was 4.8 kg, 5.5 kg, 6.3 kg, and 7.2 kg, compared with 2.8 kg with placebo and 4.1 kg with orlistat. This was 2.1 kg to 4.4 kg greater than that with placebo. Approximately 76% of people lost more than 5% of their weight with liraglutide 3.0 mg than with placebo (30%) or orlistat (44%).

Another significant observation was that liraglutide reduced blood pressure at all doses and reduced the prevalence of prediabetes (84–96% reduction) at 1.8–3.0 mg per day. Nausea and vomiting occurred more frequently in patients treated with liraglutide than in those treated with placebo. Overall, treatment with liraglutide over 20 weeks is well-tolerated, induces weight loss, improves certain obesity-related risk factors, and reduces prediabetes [71]. Another study evaluated the efficacy, safety, and use of all doses of liraglutide for weight management in obese individuals without diabetes. Nine randomized controlled trials were included, and from all doses of liraglutide for weight management in obese and non-diabetic subjects, the largest proportion of participants achieved approximately 5–10% weight loss. Trials of all doses of liraglutide concluded that the 3.0 mg dose had the most significant weight loss results [72]. Cardiovascular risk reduction was the greatest benefit reported after liraglutide administration.

Common adverse events were gastrointestinal and usually occurred early in treatment and during dose escalation. Serious adverse events, such as pancreatitis, cancer, and psychiatric effects related to all doses of liraglutide, especially the 3.0 dose, were a concern, and some individuals withdrew from the study. However, liraglutide was safe for most study participants, with minor gastrointestinal adverse events. From randomization in another study, participants on liraglutide 3.0 mg lost 7.2 kg of mean body weight at 20 weeks and 7.8 kg at one year. After two years, participants randomized to liraglutide 2.4 or 3.0 mg (pool group) sustained a mean weight loss of 5.3 kg. Those who completed the entire two-year treatment period lost 7.8 kg from the time the weight loss began at run-in. The most commonly reported side effect of liraglutide was nausea, known to be induced by supraphysiological levels of native GLP-1 and by GLP-1 RAs [73,74,75,76]. In phase 3 trials of liraglutide for T2DM and obese patients, nausea was reported in about 40% of people treated with liraglutide 1.2 or 1.8 mg daily, but it was mostly mild and transient [77,78].

### 4.3. Long-Term Follow-Up Studies

Several studies have investigated the effectiveness of glimepiride in combination with either liraglutide or rosiglitazone for treating T2DM. To evaluate the long-term clinical and economic outcomes of these treatments, we employed the CORE diabetes (Centre for Outcomes and Resource Evaluation) model, which uses epidemiological data from long-term clinical trials to simulate the morbidity, mortality, and costs of diabetes. Clinical data were obtained from the LEAD-1 study [66], which compared the effects of glimepiride with two doses of the GLP-1 analog liraglutide (1.2 mg and 1.8 mg) or rosiglitazone (4 mg) in patients with T2DM. We calibrated CORE using baseline patient characteristics from the LEAD-1 study and assessed the clinical and economic outcomes over three periods (10, 20, and 30 years), including survival rates, the cumulative incidence of cardiovascular, ophthalmic, and renal events, and the cost of medical care.

Our analysis showed that liraglutide 1.2 mg and 1.8 mg were associated with greater 30-year survival rates compared to the rosiglitazone group (15.0% and 16.0% vs. 12.6% after 30 years) and resulted in fewer cardiovascular, renal, and ocular problems in a hypothetical cohort of 5000 patients per therapy. After 30 years, the cardiovascular death rates for liraglutide 1.2 mg, 1.8 mg, and rosiglitazone were 69.7%, 68.4%, and 72.5%, respectively. Therefore, our analysis indicates that liraglutide plus glimepiride is a more effective treatment than rosiglitazone plus glimepiride in terms of projected rates of death, diabetes complications, and long-term healthcare expenditures, using data from LEAD-1 and epidemiologic evidence from the diabetes CORE model [79].

A randomized, double-blind, three-year trial (conducted from 1 June 2011 to 2 March 2015) investigated the efficacy of liraglutide in reducing the risk of diabetes and managing weight in individuals with obesity and prediabetes. The study found that liraglutide 3.0 mg may provide health benefits for people with prediabetes and obesity. However, one limitation of the study is that individuals who withdrew from treatment were not followed up [80].

### 4.4. Average Weight Loss

In terms of average weight reduction, the LEAD-1 study showed that rosiglitazone caused a significant increase in body weight (+2.1 kg, baseline 80.6 kg, *p* < 0.0001) while liraglutide 1.8 mg (−0.2 kg, baseline 83.0 kg), 1.2 mg (+0.3 kg, baseline 80.0 kg), and placebo (−0.1 kg, baseline 81.9 kg) resulted in smaller changes [65]. All liraglutide groups experienced weight loss (1.8–2.8 kg), while the glimepiride group showed weight gain (1.0 kg; *p* < 0.0001) [67].

The LEAD-4 study demonstrated that compared to placebo (0.6 +/− 0.3 kg), weight loss was achieved with doses of 1.2 and 1.8 mg of liraglutide (1.0 +/− 0.3 and 2.0 +/− 0.3 kg, respectively) [68]. Similarly, in the LEAD-5 trial, liraglutide produced more weight reduction than placebo (1.39 kg treatment difference) and glargine (3.4 kg treatment difference) [81].

When comparing liraglutide to exenatide, both drugs induced similar weight loss (liraglutide −3.24 kg vs. exenatide −2.87 kg) [70]. Evidence from a clinical trial evaluating liraglutide for weight management revealed that in addition to recommended diet and physical activity, consistent use of liraglutide resulted in a weight loss of 4 to 6 kg, with a greater proportion of patients achieving at least 5% and 10% weight loss compared to placebo. Although weight loss with liraglutide is greater than that observed with orlistat or lorcaserin, it is slightly less than that seen with phentermine/topiramate. liraglutide is effective in inducing and sustaining weight loss in obese patients, with the added benefit of improved glycemic control [82].

A study published in May 2021 focused on maintaining healthy weight loss with exercise, liraglutide, or both. The randomized, head-to-head, placebo-controlled trial involved adults with obesity (body mass index [BMI], 32 to 43) who did not have diabetes. After an eight-week low-calorie diet, participants were randomly assigned to one of four strategies: a moderate-to-vigorous exercise program plus placebo (exercise group); treatment with liraglutide (3.0 mg per day) plus usual activity (liraglutide group); exercise program plus liraglutide therapy (combined group); or placebo plus usual activity (placebo group).

The study found that 195 individuals lost an average of 13.1 kg of body weight following an eight-week low-calorie diet. At one year, all active treatment methods resulted in more weight loss than placebo: the exercise group showed a difference of 4.1 kg; the liraglutide group showed a difference of 6.8 kg; and the combined group showed a difference of 9.5 kg. The combined strategy resulted in greater weight loss than exercise (difference, −5.4 kg) but not liraglutide (−2.7 kg). The combined strategy also led to a greater decrease in body fat percentage by 3.9 percentage points, which was roughly twice that observed in the exercise group (−1.7 percentage points) and the liraglutide group (−1.9 percentage points). Improvements in insulin sensitivity, cardiorespiratory fitness, and glycosylated hemoglobin levels were only linked to the combination strategy. Exercise and liraglutide therapy together promoted healthy weight loss maintenance more than either therapy alone [83].

### 4.5. Indications and Contraindications

#### 4.5.1. Directions

Liraglutide is administered through subcutaneous injection once a day. It is an acylated glucagon-like peptide-1 analog that shares 97% amino acid homology with native glucagon-like peptide-1 and exhibits long-acting activity. liraglutide’s pharmacokinetic properties allow for 24 h glycemic management with a single dose, owing to metabolic stabilization and reduced renal filtration that slow down release from the injection site and eliminate the medication more slowly [84].

In a placebo-controlled study (Lira-1) examining the efficacy and safety of liraglutide in overweight adult patients with type 1 diabetes and poor glycemic control, there was no significant difference in HbA1c reduction between insulin plus placebo and insulin plus liraglutide treatments. However, patients who received liraglutide showed lower hypoglycemic events, bolus and total insulin doses, body weight, and increased heart rate [85]. A systematic review and meta-analysis concluded that liraglutide is effective and safe for weight loss in obese, non-diabetic individuals [86]. Furthermore, liraglutide has shown significant improvements in insulin action, cardiovascular disease (CVD) risk, weight reduction (including waist circumference), and reproductive function, including increased pregnancy rates in overweight or obese PCOS women [87].

The LEADER trial published in 2016 assessed the impact of liraglutide on cardiovascular outcomes in 9340 participants with advanced T2DM and high baseline cardiovascular risk. The primary composite outcome was first-time cardiovascular death, non-fatal myocardial infarction, or non-fatal stroke. After a median follow-up of 3.8 years, patients randomized to liraglutide experienced a substantial decline in the primary composite outcome compared to placebo, with a significant decrease in overall mortality and mortality from cardiovascular causes. In 2017, LEADER investigators reported that liraglutide therapy resulted in significantly lower nephropathy events than placebo, although there was no significant difference in retinopathy events. However, some studies suggest that liraglutide may be detrimental to patients with severe heart failure, primarily due to increased heart rate [88].

In the time-to-event analysis, the liraglutide group had fewer patients experience the primary outcome of cardiovascular death, non-fatal myocardial infarction, or non-fatal stroke than the placebo group. The average follow-up time was 3.8 years, and the liraglutide group had fewer patients die from cardiovascular reasons overall. There were no significant differences in hospitalization rates for non-fatal myocardial infarction, non-fatal stroke, and heart failure between the liraglutide and placebo groups [89].

#### 4.5.2. Contraindications and Precautions

Liraglutide’s prescribing information includes a boxed warning about the increased risk of thyroid C-cell tumors and that the medication is contraindicated in people with a personal or family history of medullary thyroid carcinoma (MTC) or multiple endocrine neoplasia syndrome type 2 (MEN 2) as well as in people with those conditions (MEN 2). Thyroid C-cell tumors have been observed in rodents but not humans [90]. Additionally, there is a warning about the potential risk of acute pancreatitis and pancreatic cancer. While these risks have not been confirmed through completed clinical trials, continued vigilance is required [91,92]. A review of amylase/lipase activity levels and acute pancreatitis events in the SCALE weight management trials found that there were reversible, dose-independent increases in amylase/lipase activity that were unrelated to baseline characteristics and did not predict the occurrence of acute pancreatitis and possibly gallstones that contributed to 50% of cases of acute pancreatitis [93].

Common side effects of liraglutide include nausea and diarrhea [94]. These and other gastrointestinal disturbances, such as vomiting, decreased appetite, indigestion, and constipation, are usually dose-related and generally occur within the first few weeks of treatment. To reduce these gastrointestinal symptoms, it is recommended to start treatment with a dose of 0.6 mg daily and increase it by 0.6 mg daily every week until reaching the daily dose of 3 mg. If the 3 mg dose cannot be tolerated, treatment should be discontinued, although some weight loss would still be expected.

There may also be a risk of acute gallbladder disease with liraglutide, but gallstones may also be related to acute weight loss. However, a study of the effects of liraglutide on gallbladder emptying in overweight or obese adults showed no effect on maximal postprandial gallbladder ejection fraction (GBEFmax), although time to GBEFmax appeared to be prolonged [95].

Hypoglycemia is likely to occur only in patients with T2DM treated with insulin or sulphonylurea. Reports suggest that liraglutide may cause a deterioration of kidney function, which may be related to nausea, vomiting, diarrhea, or dehydration. However, an exploratory post hoc analysis of pooled neuropsychiatric safety data from all randomized, double-blind phase 2 and 3a trials with liraglutide 3.0 mg showed a small numerical imbalance in suicidal ideation with liraglutide by event reporting adverse effects. However, there was no between-treatment imbalance in suicidal ideation/behavior or depression observed by prospective questionnaire assessments [96].

Liraglutide has been used in patients with varying degrees of renal impairment in some cases. liraglutide 1.8 mg daily did not affect renal function and showed better glycemic control with no increased risk of hypoglycemia compared to placebo in studies comparing its safety and efficacy as an adjunct to glucose-lowering therapy in patients with T2DM and moderate renal impairment (LIRA RENAL) with estimated glomerular filtration rate (eGFR) 30–59 mL/min/1.73 m^2^ by MDRD [97]. The Effect and Action in Diabetes (LEAD) studies also showed that the glycemic efficacy and safety of liraglutide (1.2 mg or 1.8 mg) were similar in patients with mild renal impairment (eGFR 60 to ≤89 mL /min/1.73 m^2^) to those with normal renal function [96]. In a prespecified secondary analysis from the LEADER trial, liraglutide at doses up to 1.8 mg per day added to usual care resulted in lower rates of development and progression of diabetic kidney disease than placebo [98]. No dose adjustment of liraglutide is required in patients with renal impairment. The same is true for patients with varying degrees of liver failure. A small increase in heart rate occurs with liraglutide and other long-acting GLP-1RAs. The cause and significance of this are uncertain, but it may tend to increase cardiovascular risk, particularly in relation to heart failure, although this is usually overshadowed in atherosclerotic cardiovascular disease (ASCVD) events by the reduction of other factors of cardiovascular risk. [99]. liraglutide did not significantly reduce heart failure hospitalizations in the LEADER trial [89], and there were more serious cardiac adverse events in patients with chronic heart failure with reduced left ventricular ejection fraction treated with liraglutide 1.8 mg once daily than placebo in a study of left ventricular function in patients with stable chronic heart failure with and without diabetes (LIVE) [100]. liraglutide provides a useful addition to the armamentarium of weight reduction pharmacotherapy. The 3 mg daily dose provides sustained weight reductions of approximately 4 to 6 kg more than the placebo in overweight and obese patients with and without T 2 DM. Nausea is the most common side effect, and this can be reduced by starting at a low dose and gradually increasing the dose. Serious adverse effects are uncommon, and the major limitations are cost and the need for daily injections [101]. Table 1 summarizes the clinical trials of liraglutide.

## 5. Semaglutide

Semaglutide is a T2DM medication from the class of incretino-mimetics known as GLP-1 RAs. Regardless of the existence of T2DM, the STEP trials examined semaglutide at the higher dose of 2.4 mg/week particularly to induce weight loss.

### 5.1. Weight Loss Evidence from Clinical Trials in Diabetics and Non-Diabetics

Semaglutide is a GLP-1 analog that has been studied extensively in clinical trials. The STEP-1 trial demonstrated that Semaglutide 2.4 mg once weekly, when used as a supplement to lifestyle modifications, led to a clinically significant reduction in body weight in overweight or obese patients [102]. Similarly, the STEP-2 trial found that Semaglutide 2.4 mg once weekly was more effective than semaglutide 1.0 mg or placebo for treating Type 2 diabetes mellitus (T2DM) and obesity [103].

The STEP-3 trial investigated the effects of semaglutide as a supplement to intensive behavioral therapy in overweight or obese individuals. The results indicated that semaglutide produced considerably better weight loss over 68 weeks than placebo [104]. Maintenance of weight loss over time was examined in the STEP-4 trial, which demonstrated that semaglutide leads to continuous weight loss [44]. The STEP-5 trial evaluated the effects of semaglutide in overweight or obese adults over a two-year period and found that semaglutide treatment led to significant and long-lasting weight loss [105].

In the STEP-6 trial, the effects of semaglutide were evaluated in obese East Asian adults with and without T2DM. The results showed that semaglutide 2.4 mg once weekly had superior reductions in body weight and abdominal visceral fat area compared to placebo, indicating that it is a promising treatment option for weight management in this population [106].

The STEP-7 trial has ended, but the analyzed data have not yet been published. It occurred in China, Hong Kong, the Republic of Korea, and Brazil, with participants receiving semaglutide or a placebo for 44 weeks. Another trial compared semaglutide once a week to liraglutide once a day in overweight or obese adults without diabetes. The results showed that semaglutide once a week resulted in significantly greater weight loss than liraglutide once a day [107]. Finally, treatment with semaglutide plus lifestyle intervention resulted in a greater reduction in BMI in obese adolescents compared to lifestyle intervention alone [108].

### 5.2. Semaglutide High Doses for Non-Diabetic Obese

A systematic review and meta-analysis of randomized controlled trials on the effectiveness and safety of semaglutide for weight loss in obese or overweight patients without diabetes were recently published by Gao X et al. [109]. In this meta-analysis, eight trials and a total of 4567 patients were included. Semaglutide caused a greater reduction in waist circumference (MD: −8.28 cm; 95% CI: −9.51 to −7.04; *p =* 0.00001), body mass index (MD: −3.71 kg/m^2^; 95% CI: −4.33 to −3.09; *p* = 0.00001), and weight loss of 5, 10, 15, and 20% with a greater proportion of participants when compared to placebo. Semaglutide displayed more negative side effects than placebo, mostly gastrointestinal issues. It also demonstrated a favorable effect on blood pressure, C-reactive protein, and lipid profiles. With dose dependence, the results were consistent and reliable. In obese or overweight patients without diabetes, semaglutide has demonstrated considerable weight loss with acceptable safety [110].

### 5.3. Long-Term Follow-Up Studies

A pooled analysis of two clinical trials, SUSTAIN6 and LEADER, was conducted to assess the impact of semaglutide administered once a week and liraglutide administered once daily on renal outcomes in patients with type 2 diabetes mellitus (T2DM). The follow-up period for SUSTAIN6 and LEADER were 2.1 and 3.8 years, respectively. The pooled analysis revealed that semaglutide/liraglutide reduced albuminuria from baseline to two years after randomization by 24% compared to the placebo (95% CI, 20–27%; *p* < 0.001). The trial data analysis also showed significant reductions (*p* < 0.001 for all), with the most prominent reduction observed in the semaglutide 1.0 mg group (33% [95% CI, 24–40%]; *p* < 0.001) at two years. Furthermore, semaglutide 1.0 mg and liraglutide significantly slowed down the decline in estimated glomerular filtration rate (eGFR) slope by 0.87 and 0.26 mL/min/1.73 m^2^/year (*p* < 0.0001 and *p* < 0.001), respectively, compared to the placebo. The beneficial effects of semaglutide/liraglutide were more apparent in patients with baseline eGFR < 60 compared to those with eGFR ≥ 60 mL/min/1.73 m^2^ (*p* = 0.06 and 0.008 for semaglutide 1.0 mg and liraglutide, respectively). Moreover, semaglutide/liraglutide significantly reduced the risk of persistent eGFR reductions by 40% and 50% compared to the placebo (hazard ratio [HR], 0.86 [95% CI, 0.75–0.99]; *p* = 0.039 and HR, 0.80 [95% CI, 0.66–0.99]; *p* = 0.023, respectively). Directional results were also observed for eGFR reductions of 30% and 57%, but they were not significant (HR, 0.92 [95% CI, 0.84–1.02]; *p* = 0.10 and HR, 0.89 [95% CI, 0.69–1.13]; *p* = 0.34). Patients with a baseline eGFR of 30 to <60 mL/min/1.73 m^2^ showed an increased probability of persistent eGFR reductions for all thresholds, ranging from HR 0.71 for a 30% reduction (95% CI, 0.59–0.85; *p* = 0.0003 *p* = 0.017) to 0.54 for the 57% reduction (95% CI, 0.36–0.81; *p* = 0.003 *p* = 0.035). In summary, the findings suggest that semaglutide/liraglutide provides kidney protective effects, particularly in patients with pre-existing chronic kidney disease. Another long-term study, STEP 5, compared once-weekly subcutaneous semaglutide 2.4 mg to placebo (along with behavioral treatment) for two years in individuals with obesity or overweight and at least one weight-related comorbidity but no diabetes [105].

### 5.4. Average Weight Loss

In the semaglutide treatment groups, the mean decrease in body weight from baseline to week 68 was significantly higher compared to the placebo group. Specifically, the reduction was 14.9% in the semaglutide group versus −2.4% in the placebo group [102]. Moreover, the proportion of participants who achieved a weight loss of at least 5%, 10%, and 15% was significantly greater in the semaglutide group compared to the placebo group (*p* < 0.001 for all three odds comparisons) [102]. The estimated treatment difference for semaglutide 2.4 mg versus placebo was −6.2 percentage points (95% CI −7.3 to −5.2; *p* < 0.0001) [103]. Additionally, in the STEP-3 study, semaglutide treatment resulted in a greater proportion of participants achieving weight loss of at least 10% or 15% compared to the placebo group (75.3% versus 27.0% and 55.8% versus 13.2 %; *p* < 0.001) [104].

In terms of maintenance of weight loss, continued semaglutide treatment demonstrated a mean change in body weight from week 20 to week 68 of −7.9%, compared to +6.9% in the placebo group (difference, −14.8 [95% CI, −16.0 to −13.5] percentage points; *p* < 0.001) [43]. Moreover, in the STEP-5 study, the average change in body weight from baseline to week 104 was −15.2% in the semaglutide group compared to −2.6% in the placebo group (estimated treatment difference, −12.6% points; 95% confidence interval, from −15.3 to −9.8; *p* < 0.0001) [105]. Notably, more participants in the semaglutide group achieved at least 5% weight loss from baseline at week 104 compared to the placebo group (77.1% versus 34.4%; *p* < 0.0001) [105].

Furthermore, cardiometabolic risk variables and participant-reported physical functioning improved significantly more in semaglutide-treated participants compared to placebo-treated participants [102]. Waist circumference, systolic blood pressure, and SF-36 physical functioning score also improved with continuous subcutaneous semaglutide compared to placebo (all *p* < 0.001) [43].

In a study conducted on obese East Asian adults, with or without T2DM, who received semaglutide 2.4 mg once weekly, the estimated mean change in body weight from baseline to week 68 was −13.2% (SEM 0.5) in the semaglutide 2.4 mg group, and −9.6% (0.8) in the semaglutide 1.7 mg group, compared to −2.1% (0.8) in the placebo group (estimated treatment difference [ETD] −11.1 percentage points [95% CI −12.9 to −9.2] for semaglutide 2.4 mg versus placebo; −7.5 percentage points [95% CI −9.6 to −5.4] for semaglutide 1.7 mg versus placebo; both *p* < 0.0001). At week 68, a greater proportion of participants achieved a 5% or greater reduction in baseline weight in the semaglutide 2.4 mg group (160 [83%] of 193 participants) and the semaglutide 1.7 mg group (71 [72%] of 98 participants) than in the placebo group (21 [21%] of 100 participants); odds ratio [OR] 21.7 [95% CI 11.3 to 41.9] for semaglutide 2.4 mg versus placebo; OR 11.1 [95% CI 5.5 to 22.2] for semaglutide 1.7 mg versus placebo; both *p* < 0.0001). The abdominal visceral fat area was reduced by 40.0% (SEM 2.6) among participants in the semaglutide 2.4 mg group and by 22.2% (3.7) among participants in the semaglutide 1.7 mg group, compared to 6.9% (3.8) in the placebo group (ETD −33.2% [95% CI −42.1 to −24.2] for semaglutide 2.4 mg versus placebo; −15.3% [95% CI −25.6 to −4.9] for semaglutide 1.7 mg versus placebo) [106].

In the comparison of semaglutide once weekly versus liraglutide daily (STEP-8), the mean weight change from baseline was −15.8% with semaglutide versus −6.4% with liraglutide (difference, −9.4 percentage points [95% CI, −12.0 to −6.8]; *p* < 0.001); weight change with combined placebo was −1.9%. Participants were significantly more likely to achieve weight loss of 10% or more, 15% or more, and 20% or more with semaglutide versus liraglutide (70.9% of participants versus 25.6% [odds ratio, 6.3 {95% CI, 3.5 to 11.2}], 55.6% versus 12.0% [odds ratio, 7.9 {95% CI, 4.1 to 15.4}] and 38.5% vs. 6.0%, respectively [odds ratio, 8.2 {95% CI, 3.5 to 19.1}], all *p* < 0.001) [107]. Overall, these findings demonstrate the efficacy of semaglutide in promoting weight loss and improving cardiometabolic risk factors, which may have implications for the management of obesity and related disorders.

The STEP-TEENS study is a noteworthy example of research on weight loss in obese teenagers. Semaglutide demonstrated a significant decrease in BMI, with a mean reduction of −16.1% from baseline to week 68, compared to the placebo group. The estimated difference was −16.7 percentage points, with a 95% confidence interval [CI] of −20.3 to −13.2 and a *p*-value of 0.001. Moreover, 73% (95 out of 131 participants) in the semaglutide group had lost at least 5% of their body weight by week 68, whereas only 18% (11 out of 62 participants) in the placebo group had achieved this result. The ratio of estimated odds was 14.0, with a 95% CI of 6.3 to 31.0 and a *p*-value of <0.001. In addition, semaglutide was found to be more effective than the placebo in reducing body weight and improving cardiometabolic risk variables such as waist circumference, glycated hemoglobin, lipids (except high-density lipoprotein cholesterol), and alanine aminotransferase levels [108]. The study’s results provide valuable insights into potential treatments for obesity in teenagers, although further research is necessary to confirm these findings and investigate any potential long-term effects.

### 5.5. Indications and Contraindications

Oral semaglutide obtained FDA approval in September 2019 to enhance glycemic control in individuals with T2DM. The product comes in tablets of 3, 7, and 14 mg. The oral semaglutide dosage should be increased to 7 mg once daily after 30 days, according to the manufacturer, who suggests starting with 3 mg once daily. After at least 30 days on the 7 mg dose, the dose can be further increased to 14 mg once daily for patients who need further blood glucose lowering. For a second indication of reducing major adverse cardiovascular events (MACE) in people with T2DM and existing cardiovascular disease, the firm has submitted an application to the FDA. In accordance with a company news statement, the FDA review for the MACE indication is anticipated to be finished in the first quarter of 2020 [103,111]. Subcutaneous semaglutide is indicated in the once-weekly treatment of adult patients with T2DM to improve glycemic control, in combination with (1) diet and exercise in patients for whom metformin is inappropriate due to contraindication or intolerance; (2) metformin, when diet and exercise plus the maximum tolerated dose of metformin do not achieve adequate glycemic control; (3) metformin and a sulphonylurea (SU), when diet and exercise plus metformin and SU dual therapy do not achieve adequate glycemic control; and (4) basal insulin with metformin, when diet and exercise plus basal insulin with metformin do not achieve adequate glycemic control [112].

Semaglutide should be indicated with caution in the following conditions: diabetic retinopathy, a type of eye damage due to diabetes, hypoglycemia, gallbladder dysfunction, acute pancreatitis, chronic kidney disease with low GFR, medullary thyroid cancer, and multiple endocrine neoplasias.

GLP1-RAs were linked to a higher risk of pancreatitis and pancreatic cancer, according to a meta-analysis published in September 2017 [113]. However, those meta-analyses did not include recently published cardiovascular outcome studies (CVOT) with GLP1- RAs, which give a large additional body of data. Previous meta-analyses of randomized controlled trials have failed to demonstrate any appreciable increase in risk. The current meta-analysis aims to assess GLP1-RA’s impact on cholelithiasis, pancreatic cancer, and pancreatitis. Thirteen of the 113 studies that fulfilled the inclusion criteria provided no information on pancreatitis, and 72 of the studies that met the criteria reported no incident in any of the therapy groups. GLP1-RAs did not substantially raise the risk of cholelithiasis (MH-OR [95% CI] 1.30 [1.01–1.68], *p* = 0.041), while they did not significantly increase the incidence of pancreatitis or pancreatic cancer (MH-OR [95% CI] 0.93 [0.65–1.34], *p* = 0.71 and 0.94 [0.52–1.70], *p* = 0.84, respectively). So, based on the information that is now available, GLP-1 RAs are safe for use in treating pancreatitis. In contrast, therapy with these medications is linked to a higher incidence of cholelithiasis, which warrants more research [113].

Another meta-analysis compared the benefits and harms of blood glucose-lowering drugs in adults with T2DM. This included 453 studies evaluating 21 antidiabetic interventions from 9 drug classes. It was stated that subcutaneous semaglutide and canagliflozin increased diabetic retinopathy and amputation, respectively [114].

Semaglutide has also demonstrated efficacy in reducing liver injury indices and liver fat content. Multiple studies indicate that these drugs are able to promote the resolution of steatohepatitis in a significant proportion of patients with NASH and to reduce the progression of hepatic fibrosis [115,116].

### 5.6. Adverse Effects

The results of the STEP-1 trial demonstrated that the most frequently reported adverse effects associated with semaglutide were nausea and diarrhea, which were generally temporary, mild to moderate in severity, and improved over time. However, due to gastrointestinal issues, a higher number of study participants in the semaglutide group (59 [4.5%] vs. 5 [0.8%]) withdrew from the study.

In the STEP-2 trial [103], adverse events were more commonly observed in patients treated with semaglutide 2.4 mg (353 [87.6%] out of 403 patients) and 1.0 mg (329 [81.8%] out of 402 patients) compared to those receiving placebo (309 [76.9%] out of 402 patients). Furthermore, semaglutide showed a higher incidence of gastrointestinal side effects in the STEP-3 trial (82.8%) compared to placebo (63.2%) [104]. In the same trial, 49.1% of participants treated with subcutaneous semaglutide experienced gastrointestinal events, compared to 26.1% of those receiving placebo; a similar proportion of patients discontinued treatment due to adverse events in both groups (2.4% for semaglutide vs. 2.2% for placebo) [43].

In the STEP-5 clinical program, after a two-year follow-up, gastrointestinal adverse events were more frequently reported with semaglutide than with placebo (82.2% vs. 53.9%) [105]. In the STEP-6 trial, mild to moderate gastrointestinal disturbances were reported by 118 (59%) out of 199 participants in the semaglutide 2.4 mg group, 64 (64%) out of 100 participants in the semaglutide 1.7 mg group, and 30 (30%) out of 101 participants in the placebo group. Adverse events leading to discontinuation of the study product occurred in 5 (3%) out of 199 participants in the semaglutide 2.4 mg group, 3 (3%) out of 100 participants in the semaglutide 1.7 mg group, and 1 (1%) out of 101 participants in the placebo group [106]. Gastrointestinal adverse events were also more common in the semaglutide vs. liraglutide comparison (84.1% vs. 82.7%) [107].

In adolescents, the incidence of gastrointestinal adverse events was higher with semaglutide than with placebo (62% vs. 42%). Five participants (4%) in the semaglutide group and no participants in the placebo group had cholelithiasis. Serious adverse events were reported in 15 out of 133 participants (11%) in the semaglutide group and 6 out of 67 participants (9%) in the placebo group [108]. Table 2 summarizes the clinical trials of semaglutide.

## 6. Tirzepatide

### 6.1. Studies Completed on Tirzepatide

Currently, there have been eight completed studies related to tirzepatide, a new agonist of GIP and GLP-1 receptors. The first study, SURPASS-1, was conducted from 3 June 2019 to October 2020 across 52 medical research centers and hospitals in India, Japan, Mexico, and the USA. The study evaluated the efficacy, safety, and tolerability of tirzepatide, a double-dependent insulinotropic polypeptide of glucose and the GLP-1 receptor agonist, as a monotherapy compared to placebo in people with T2DM who had inadequate control with diet and exercise [117].

The second study, SURPASS-2, compared the efficacy and safety of once-weekly tirzepatide to semaglutide, a selective GLP-1 RA. The primary endpoint of this study was the change in glycated hemoglobin levels from baseline to 40 weeks [118].

In the third study, SURPASS-3, researchers evaluated the efficacy and safety of tirzepatide compared to titrated insulin degludec in people with T2DM who had inadequate control of metformin with or without SGLT2 inhibitors. This open-label, parallel-group study was conducted in 122 sites across 13 countries between 1 April and 15 November 2019, with 1947 participants assessed for eligibility and 1444 randomized to treatment [119].

The SURPASS-4 study investigated the efficacy and safety, with a focus on cardiovascular safety, of the novel dual GIP and GLP-1 RA tirzepatide compared to insulin glargine in adults with T2DM and high cardiovascular risk who had inadequate control with oral antidiabetic agents. The study was conducted in 187 locations across 14 countries on five continents, with 3045 participants examined between 20 November 2018 and 30 December 2019 [120].

In the SURPASS-5 phase 3 randomized clinical trial, researchers evaluated the efficacy and safety of tirzepatide in 475 patients with T2DM who had insufficient glycemic control while receiving once-daily insulin glargine treatment, with or without metformin, at 45 medical research facilities and hospitals in eight countries. Patients were enrolled on 30 August 2019 and monitored until 13 January 2021. The primary outcome was the glycated hemoglobin A1c (HbA1c) level at week 40 and its mean change from baseline. The study also measured other important secondary endpoints, including the average change in body weight and the proportion of patients whose HbA1c levels were within the predetermined ranges [121].

SURPASS J-mono evaluated the efficacy and safety of tirzepatide compared to dulaglutide in Japanese patients with T2DM. The study was conducted in 46 medical research centers and hospitals in Japan, with 821 participants assessed for study eligibility between 7 May 2019 and 31 March 2021. A total of 636 participants were randomly assigned to receive at least one dose of tirzepatide (5 mg, 10 mg, or 15 mg) or dulaglutide (0.75 mg), with 615 participants (97%) completing the study, and 21 (3%) discontinuing the study [122]. Finally, the safety and glycemic efficacy of tirzepatide as adjunctive treatment in Japanese patients with T2DM who had inadequate glycemic control with stable doses of various oral antihyperglycemic monotherapies were investigated in the SURPASS J-combo study. The study was conducted in 34 medical research centers and hospitals in Japan. Eligible participants were 20 years of age or older with inadequately controlled T2DM (HbA 1c ≥ 7.0% to <11.0%) and were receiving oral antihyperglycemic monotherapy (sulfonylurea, biguanides, α-glucosidase inhibitors, thiazolidinedione, glinides, or SGLT2 inhibitors) for at least three months. The primary endpoint was safety and tolerability over 52 weeks of treatment, assessed as the incidence of treatment-emergent adverse events in the intention-to-treat population [123].

### 6.2. Average Weight Loss

The results of the first SURPASS-1 study demonstrated a dose-dependent weight loss of 7 to 9.5 kg [117]. Similarly, the SURPASS-2 Study showed that reductions in body weight with tirzepatide were dose-dependent. After 40 weeks, the mean body weight reductions with tirzepatide were −7.6 kg, −9.3 kg, and −11.2 kg at 5 mg, 10 mg, and 15 mg, respectively, compared to −5.7 kg with semaglutide. At all doses, tirzepatide was found to be superior to semaglutide [118]. Notably, tirzepatide led to significant weight loss at all three doses (from −7.5 kg to −12.9 kg) in week 52, whereas insulin degludec resulted in an increase in body weight by 2.3 kg [119]. Furthermore, the mean change in body weight from baseline was −5.4 kg with 5 mg of tirzepatide, −7.5 kg with 10 mg, −8.8 kg with 15 mg, and 1.6 kg with placebo in the SURPASS 5 study [121]. A mean difference of at least −5.8 kg for 5 mg, −8.5 kg for 10 mg, and −10.7 kg for 15 mg of tirzepatide was observed compared to −0.5 kg for dulaglutide, indicating that tirzepatide was associated with dose-dependent reductions in body weight [122].

In the SURPASS-J combination study, the mean decreases in body weight from baseline was −3.8 kg in the 5 mg group, −7.5 kg in the 10 mg group, and −10.2 kg in the 15 mg group at week 52 [123].

SURMOUNT-1, the most comprehensive study on patients with obesity and diabetes, included 2539 adults with an average body weight of 104.8 kg and an average BMI of 38.0. Furthermore, 94.5% of participants had a BMI of 30 or more. At week 72, the mean percent change in weight was −15.0% with weekly doses of 5 mg tirzepatide, −19.5% with doses of 10 mg, −20.9% with doses of 15 mg, and −3.1% with placebo. The percentage of participants who had a weight loss of 5% or more was 85% with 5 mg, 89% with 10 mg, and 91% with 15 mg of tirzepatide, whereas only 35% of participants experienced this weight loss with a placebo. Furthermore, 50% and 57% of participants in the 10 mg and 15 mg groups, respectively, had a reduction in body weight of 20% or more, compared to only 3% in the placebo group [124].

### 6.3. Indications and Contraindications

Tirzepatide is an FDA-approved medication for the treatment of T2DM. It is a dual agonist of glucose-dependent insulinotropic polypeptide (GIP) and glucagon-like peptide-1 (GLP-1). However, it is important to note that tirzepatide has not been studied in individuals with pancreatitis and is not approved for the treatment of type 1 diabetes. tirzepatide is administered as a once-weekly subcutaneous injection with gradual dose increases and has been shown to significantly improve glycemic control and induce weight loss in T2DM patients. It is currently used as a second-line drug for diabetes, similar to other GLP-1 drugs like semaglutide, and may also be used off-label to treat obesity.

Current clinical data has demonstrated that tirzepatide is superior to placebo in improving hemoglobin A1C levels in T2DM patients. The SURPASS-5 clinical trial showed a reduction of −2.11% in hemoglobin A1C levels with 5 mg of tirzepatide per week compared to −0.86% with placebo. At the highest dose of 15 mg per week, tirzepatide resulted in a reduction of −2.34% in hemoglobin A1C levels, observed over 40 weeks. Additionally, a dose-dependent correlation was observed between weight loss and tirzepatide dosage, with a weight loss of 5.4 kg observed with 5 mg of tirzepatide and a reduction of 10.5 kg with 15 mg, similar to semaglutide. Tirzepatide has demonstrated greater efficacy compared to GLP-1 drugs in improving glycemic control and inducing weight loss.

Tirzepatide is likely to play an indirect role in treating non-alcoholic fatty liver disease (NAFLD) due to its weight loss properties and lack of liver toxicity. However, further research is needed before it can be approved for NAFLD [115,116]. Phase III studies of tirzepatide are ongoing to evaluate its potential cardiovascular benefits and support indications for patients with T2DM and obesity.

Contraindications: Animal studies have shown the potential for developing medullary thyroid carcinoma with tirzepatide. It is currently not known if this risk also applies to humans. Therefore, tirzepatide should be avoided in individuals with a personal or family history of medullary thyroid carcinoma or multiple endocrine neoplasia type 2 (MEN 2). Patients with other risk factors for thyroid cancer should also be advised of the theoretical risks. Additionally, patients who experience hypersensitivity reactions should avoid further use of tirzepatide. Other relative contraindications include gallbladder disease and diabetic retinopathy.

Tirzepatide is approved only for use in patients with T2DM and should not be used in individuals with type 1 diabetes or other forms of diabetes, such as latent autoimmune diabetes in adults. Patients currently using other GLP-1 agents, such as semaglutide or liraglutide, should not be prescribed tirzepatide. Patients on insulin therapy may be initiated on tirzepatide therapy and cautiously tapered to minimize the risk of hypoglycemia [125].

### 6.4. Adverse Effects

The most common adverse events associated with tirzepatide were mild to moderate and transient gastrointestinal events, including nausea (12–18% vs. 6%), diarrhea (12–14% vs. 8%), and vomiting (2–6% vs. 2%). No clinically significant (<54 mg/dL [<3 mmol/L]) or severe hypoglycemia has been reported with tirzepatide in SURPASS-1 clinical study [117].

In the SURPASS-2 study (tirzepatide versus semaglutide), the most common adverse events were gastrointestinal and were primarily mild to moderate in severity in the tirzepatide and semaglutide groups (nausea, 17 to 22% and 18%; diarrhea, 13 to 16% and 12%; and vomiting, 6 to 10% and 8%, respectively). Serious adverse events were reported in 5 to 7% of patients receiving tirzepatide and 3% of those receiving semaglutide [118].

The SURPASS-3 study, like the other studies previously mentioned, showed the same mild to moderate gastrointestinal adverse events, which decreased over time. A higher incidence of nausea (12–24%), diarrhea (15–17%), decreased appetite (6 12%), and vomiting (6–10%) was reported in participants treated with tirzepatide than in those treated with insulin degludec (2%, 4%, 1%, 1%, respectively). Hypoglycemia (<54 mg/dL or severe) was reported in five (1%) patients at the 5 mg dose, four (1%) at the 10 mg dose, and eight (2%) at the 15 mg dose, compared with 26 (7%) to insulin degludec. Discontinuation due to an adverse event was more common in the tirzepatide groups than in the insulin degludec group. Five participants died during the study; none of the deaths were considered by the investigators to be related to the study treatment [119]. Regarding the comparison of tirzepatide vs. insulin glargine (SURPASS-4), we observe the following aspects: nausea (12–23%), diarrhea (13–22%), decreased appetite (9–11%) and vomiting (5–9%) were more common with tirzepatide than with glargine (nausea 2%, diarrhea 4%, low; appetite <1%, and vomiting 2%, respectively); most cases were mild to moderate and occurred during the dose escalation phase. The percentage of participants with hypoglycemia (glucose < 54 mg/dL or severe) was lower with tirzepatide (6–9%) versus glargine (19%), particularly in participants not taking a sulfonylurea (tirzepatide 1–3% vs. glargine 16%). Adjudicated MACE-4 events (cardiovascular death, myocardial infarction, stroke, hospitalization for unstable angina) occurred in 109 participants and were not increased with tirzepatide compared with glargine (hazard ratio 0.74, 95% CI 0.51–1.08). Sixty deaths (*n* = 25 [3%] tirzepatide; *n* = 35 [4%] glargine) occurred during the study [120].

In the tirzepatide versus placebo groups, diarrhea (12–21% vs. 10%) and nausea (13–18% vs. 3%) were the most frequent treatment-emergent side events (SURPASS-5) [121]. And in the SURPASS-J mono study, the most frequent adverse events were gastrointestinal (23 [4%] of 636): nausea (19 [12%] participants in the 5 mg group vs. 31 [20%] in the 10 mg vs. 32 [20%] in the 15 mg group, all who received tirzepatide vs. 12 (8%) in the dulaglutide group), and constipation (24 [15%] vs. 28 [18%] vs. 22 [14 %] vs. 17 [11%]) Another adverse symptom was nasopharyngitis (29 [18%] vs. 25 [16%] vs. 22 [14%] vs. 26 [16%]) [122].

In the SURPASS-J combo study, the majority of participants (343 [77%] of 443) had at least one treatment-emergent adverse event. This was more common in the tirzepatide 15 mg group (125 [84%] of 148) than in the 5 mg (109 [74%] of 148) and 10 mg (109 [74%] of 148) groups 147). The most common adverse events with tirzepatide were mild or moderate nasopharyngitis (75 [17%]), nausea (74 [17%]), constipation (54 [12%]), diarrhea (51 [12%]), and decreased appetite (44 [10%]) [123].

Finally, in SURMOUNT-1 study, the most common adverse events associated with tirzepatide were gastrointestinal, and most were mild to moderate in severity, occurring mainly during dose escalation. Adverse events led to treatment discontinuation in 4.3%, 7.1%, 6.2%, and 2.6% of participants who received tirzepatide 5 mg, 10 mg, and 15 mg doses of placebo [122,124]. Table 3 summarizes the clinical trials of tirzepatide.

## 7. Lixisenatide and Exenatide-STUDIES and Evidence

### 7.1. Lixisenatide

Lixisenatide is an incretin mimetic, a type of GLP-1 RA, used for treating T2DM through subcutaneous injection. The clinical trials for Lixisenatide approval are known as GetGoal trials. The oldest of these trials is GetGoal MONO, which assessed the effectiveness and safety of Lixisenatide monotherapy in T2DM. In this 12-week trial, Lixisenatide at a dose of 20 μg resulted in a significant mean reduction of 0.85% in glycated hemoglobin (HbA1c), compared to a 0.19% reduction in the placebo group. It also showed a significant decrease of 75% in glucose excursion and was well tolerated [125,126].

Another study, GETGOAL-M, aimed to evaluate the efficacy and safety of Lixisenatide (20 μg once daily, administered before the morning or evening meal) in patients with T2DM who were insufficiently controlled with metformin monotherapy. The 24-week study included 680 patients, and the morning dose of Lixisenatide significantly reduced blood glucose 2 h postprandial compared to placebo (mean change −5.9 vs. −1.4 mmol/L). The mean difference in fasting blood glucose was significant in both the morning (−0.9 mmol/L, *p* < 0.0001) and evening (−0.6 mmol/L, *p* = 0.0046) groups versus placebo. The trial confirmed that Lixisenatide improves glycemic control, has a pronounced postprandial effect, and is well-tolerated [127].

In the GETGOAL X trial, the efficacy and safety of once-daily Lixisenatide were compared with twice-daily exenatide in T2DM patients inadequately controlled with metformin. The 24-week, open-label, parallel-group, multicenter study found that add-on Lixisenatide once daily showed non-inferior improvements in HbA1c compared to exenatide twice a day, with a slightly lower mean weight loss, a lower incidence of hypoglycemia, and better gastrointestinal tolerability [128].

The GetGoal-F1 trial evaluated the efficacy and safety of once-daily Lixisenatide dose-escalation regimens in people with T2DM who had not been effectively managed by metformin. One- or two-step Lixisenatide dose-escalation regimens significantly improved glycemic control and decreased body weight over 24 weeks and a long-term extension period without increasing hypoglycemia. The study confirmed that tolerability in the one-stage group was at least similar to two-stage escalation, with the frequency of nausea/vomiting and hypoglycemia being lower in the one-stage regimen [129].

The GetGoal-S trial showed that Lixisenatide reduced HbA1c and body weight in people with poor glucose control on metformin plus sulphonylurea. Lixisenatide significantly reduced HbA1c at week 24 compared to the placebo and helped more patients achieve HbA1c < 7.0%. Lixisenatide did not significantly increase symptomatic hypoglycemia compared to placebo [130].

The GetGoal-P trial investigated the effectiveness and safety of once-daily prandial Lixisenatide compared to placebo in patients with type 2 diabetes mellitus (T2DM) who were insufficiently controlled by pioglitazone ± metformin. A total of 484 patients were randomized, with 323 patients receiving Lixisenatide and 161 receiving a placebo. After 24 weeks of treatment, Lixisenatide significantly improved glycemic control by reducing HbA1c by 0.56% compared to 0.26% in the placebo group. In addition, fasting plasma glucose was significantly improved by −0.84 mmol/L compared to placebo [131].

The GetGoal-L study was a 24-week randomized comparison of once-daily Lixisenatide versus placebo in T2DM patients who were inadequately controlled by established basal insulin. The primary endpoint was the reduction in HbA1c from baseline. Lixisenatide reduced HbA1c by an average of 0.7% over 24 weeks, significantly greater than the average reduction of 0.4% achieved in the placebo group [132].

When oral therapy for T2DM is ineffective, the addition of basal insulin is commonly used to improve glycemic control. However, when HbA1c remains elevated due to postprandial hyperglycemia, the next therapeutic step is controversial. In the GetGoal-Duo 1 study, conducted in 2009, the efficacy and safety of Lixisenatide in patients with HbA1c still elevated after initiation of insulin glargine was examined. It was found that the addition of Lixisenatide reduced HbA1c by 0.71% versus 0.40% with a placebo. More participants achieved HbA1c < 7% with Lixisenatide (56 vs. 39%). Thus, the addition of Lixisenatide to insulin glargine improved overall and post-prandial hyperglycemia and is worth considering as an alternative to prandial insulin for patients who do not achieve HbA1c goals with newly initiated basal insulin [132].

Building on the findings of GetGoal-Duo 1, the GetGoal-Duo2 trial compared Lixisenatide against a once- or three-times-daily mealtime insulin in patients on titrated basal insulin. Patients were randomized to receive once-daily Lixisenatide or once- or three-times-daily insulin glulisine added to glargine, with or without metformin. The study found that HbA1c decreased by an average of 0.6% with Lixisenatide, 0.6% with once-daily glulisine, and 0.8% with glulisine thrice-daily, making Lixisenatide non-inferior to insulin. Therefore, Lixisenatide as an add-on to basal insulin may become a preferred treatment intensification option, achieving significant glycemic targets with fewer hypoglycemic events without weight gain compared to basal-plus or basal-bolus in inadequately controlled insulin-treated patients [133,134].

The last study in the GetGoals series, initiated in 2013, examined the efficacy and safety of Lixisenatide in individuals aged 70 years or older with T2DM that was uncontrolled with their current antidiabetic treatment. A total of 350 patients were randomly assigned to either receive Lixisenatide or a placebo. Lixisenatide demonstrated a significant reduction in HbA 1c levels (−0.57% [6.2 mmol/mol]) compared to placebo (+0.06% [0.7 mmol/mol]) from baseline to week 24. Moreover, Lixisenatide was found to be more effective in reducing body weight (−1.47 kg) when compared to placebo (−0.16 kg; *p* < 0.0001). Hypoglycemia was reported in 17.6% of Lixisenatide patients compared to 10.3% of placebo recipients. In conclusion, this study supports the effectiveness of Lixisenatide over placebo in reducing HbA 1c and targeting postprandial hyperglycemia in elderly patients with inadequate glycemic control on their current antidiabetic therapy [135]. Table 4 summarizes the clinical trials of Lixisenatide.

### 7.2. Exenatide

Exenatide is the first long-acting injectable GLP-1 RA approved for treating people with T2DM at a dose of 2.0 mg/week. Its effectiveness was evaluated through the DURATION phase 3 clinical trials.

In 2008, the first study in the DURATION series, called DURATION-1, aimed to investigate the glycemic efficacy, effects on cardiovascular risk factors, and safety of exenatide once a week in patients with T2DM for over seven years. Patients were initially randomized to receive exenatide 2 mg or exenatide twice daily for 30 weeks, followed by open-label treatment with exenatide 2 mg for up to seven years. Efficacy analyses included changes from baseline in glycated hemoglobin (HbA1C) and cardiovascular risk factors. Of 295 patients in the intent-to-treat population, 122 (41%) completed seven years of treatment. Patients in the full seven-year population showed sustained improvements in blood glucose from baseline (HbA1C, 1.53%) and significant improvements in several cardiovascular risk factors, including body weight, diastolic blood pressure, total cholesterol, and cholesterol with low-density lipoprotein and high-density lipoprotein cholesterol [138].

The DURATION-2 trial demonstrated the effectiveness and safety of switching from maximum daily sitagliptin or pioglitazone to once-weekly exenatide. At 52 weeks, exenatide-treated evaluable individuals showed significant reductions in HbA1c (−1.6 ± 0.1%), fasting blood glucose (−1.8 ± 0.3 mmol/l), and weight (−1.8 ± 0.5kg). Evaluable patients who switched from once-weekly sitagliptin to exenatide showed substantial improvements in HbA1c (−0.3 ± 0.1%), fasting blood glucose (−0.7 ± 0.2 mmol/L), and weight (−1.1 ± 0.3 kg). Similarly, patients who transitioned from pioglitazone to exenatide once weekly sustained improvements in HbA1c and fasting blood glucose, along with significant weight loss (−3.0 ± 0.3 kg). Exenatide given once weekly was typically well tolerated, and the majority of adverse reactions were mild to moderate in severity. Patients who switched from daily sitagliptin or pioglitazone to once-weekly exenatide experienced better or sustained glycemic control and weight loss [139].

The DURATION-3 trial compared once-weekly exenatide to titrated insulin glargine in terms of safety and effectiveness in individuals with T2DM over the course of 84 weeks. Of the 415 patients who completed the 26-week course, 390 (194 EQW patients and 196 IG patients) enrolled in the extension trial. At 84 weeks, EQW’s A1C dropped by 1.2% compared to IG’s 1.0% from the baseline (8.3%) (*p* = 0.029). The A1C endpoint objectives of 7.0 and 6.5% were met by 44.6% of EQW patients compared to 36.8% of IG patients (*p* = 0.084) and 31.3% of EQW patients compared to 20.2% of IG patients (*p* = 0.009), respectively. It was observed that patients who received exenatide once weekly (EQW) lost 2.1 kg of body weight, whereas those who received insulin glargine (IG) gained 2.4 kg (*p* < 0.001) [134]. Additionally, the incidence of mild hypoglycemia was significantly lower in patients receiving EQW treatment (24%) in combination with metformin and sulfonylurea, as compared to IG patients (54%) (*p* < 0.001). The incidence of mild hypoglycemia in patients receiving EQW treatment and metformin alone was 8%, whereas that in IG patients was 32% (*p* < 0.001). EQW treatment also led to sustained weight loss, improved glycemic control, and a decreased risk of hypoglycemia after 84 weeks [140].

The DURATION-4 trial compared the effects of EQW to those of metformin, pioglitazone, and sitagliptin over 26 weeks in individuals with type 2 diabetes who were not receiving optimal care (i.e., diet and exercise). Patients were randomly assigned to receive SITA 100 mg/day + placebo subcutaneous (SC) (*n* = 163), IOP 45 mg/day + placebo SC (*n* = 163), MET 2000 mg/day + placebo SC (*n* = 246), EQW 2.0 mg SC + oral placebo (*n* = 248), or IOP 2000 mg/day + placebo SC (*n* = 246). After 26 weeks, the HbA1c (%) reductions with EQW were −1.53, −1.63, and −1.15, as compared to MET, IOP, and SITA, respectively. These reductions were statistically significant for IOP and SITA but not for MET. EQW treatment also led to weight loss, similar to MET, and a decreased risk of hypoglycemia. EQW and MET provided similar improvements in glycemic control and were associated with weight loss and no increased risk of hypoglycemia [141].

The DURATION-5 trial investigated the effects of once-weekly (ExQW) versus twice-daily (ExBID) glucagon-like peptide-1 receptor agonism for the treatment of patients with type 2 diabetes. The trial aimed to compare the effects of ExQW and ExBID on glycemic control, body weight, and safety over 24 weeks. The trial was conducted at 43 sites in the United States, and patients received either ExQW 2 mg for 24 weeks or ExBID 5 μg for 4 weeks, followed by ExBID 10 μg for 20 weeks. At 24 weeks, ExQW resulted in significantly greater reductions in HbA1c levels than ExBID (−1.6% vs. −0.5%, least squares mean SE, *p* = 0.0008). Both groups experienced comparable drops in mean body weight (−2.3 kg and −1.4 kg) from baseline to week 24. Patients in both groups tolerated the treatments well, with transient and predominantly mild to moderate nausea being the most common adverse event. The incidence of nausea was less frequent with ExQW (14%) than with ExBID (35%). Injection site reactions were rare but more common with ExQW. No major hypoglycemia occurred. Therefore, ExQW was found to result in superior glycemic control with less nausea than ExBID in patients with type 2 diabetes. Both groups also experienced weight loss [142].

The non-inferiority of weekly exenatide compared to daily injectable liraglutide over a 26-week treatment period was not established in a study involving 911 participants. The DURATION-6 trial compared these two GLP-1 receptor agonists and found that patients receiving liraglutide had a greater reduction in HbA (1c) (−1.48%, SE 0.05; *n* = 386) compared to those receiving exenatide (−1.28%, 0.05; 390). However, the treatment difference failed to meet predetermined non-inferiority standards (upper limit of CI 0.25%; 0.21%, 95% CI 0.08–0.33). Both liraglutide and exenatide once-weekly formulations showed improvements in glycemic control, with liraglutide resulting in greater reductions. These findings, combined with differences in injection frequency and tolerability, may help guide therapeutic decisions for T2DM patients [143].

The DURATION-7 study aimed to investigate the efficacy and safety of adding once-weekly exenatide 2 mg or placebo to insulin glargine titration (IG) ± metformin in patients with T2DM who had inadequate glycemic control. Out of 464 randomized patients, 91% completed the 28-week study. Exenatide treatment resulted in an average HbA1c reduction of 0.73% compared to placebo, with 32.5% of exenatide-treated patients achieving an HbA1c level of less than 7.0%, compared to only 7.4% in the placebo group. Body weight was reduced by an average of 1.5 kg with exenatide compared to placebo [144].

In the DURATION-NEO-1 trial, which followed patients with T2DM for 52 weeks, transitioning from twice-daily exenatide to once-weekly self-injectable exenatide suspension led to further improvement in glycemic control. Patients who made the switch experienced additional A1C reductions of approximately 0.5% (mean A1C change from baseline was −1.4% at week 52) and sustained reductions in fasting plasma glucose levels from weeks 28 to 52. Patients who continued on exenatide QWS-AI therapy for 52 weeks maintained their A1C levels and weight loss without any additional safety or tolerability issues [144].

The final study in the presented series was the DURATION-NEO-2 study, which demonstrated the efficacy and safety of a once-weekly self-injected exenatide suspension compared to sitagliptin or metformin placebo in patients with type 2 diabetes mellitus (T2DM). This was an open-label, multicenter study involving 365 patients with T2DM who had suboptimal glycemic control on metformin monotherapy. Patients were randomized to receive either exenatide 2.0 mg QWS-AI, sitagliptin 100 mg once daily, or an oral placebo. The primary endpoint was the change in glycated hemoglobin (HbA1c) from baseline to 28 weeks.

At 28 weeks, exenatide QWS-AI significantly reduced HbA1c from baseline compared to sitagliptin (−1.13% vs. −0.75% [baseline, 8.42%, and 8.50%, respectively]; *p* = 0.02) and placebo (−0.40% [baseline value, 8.50%]; *p* = 0.001). A greater proportion of patients treated with exenatide QWS-AI achieved HbA1c < 7.0% compared to patients treated with sitagliptin or placebo (43.1% vs. 32.0% and 24.6%, both *p* < 0.05). Exenatide QWS-AI and sitagliptin reduced fasting plasma glucose from baseline to 28 weeks (−21.3 and −11.3 mg/dL) compared to placebo (+9.6 mg/dL), without any significant differences between the two active treatments. Body weight decreased with both active treatments (−1.12 and −1.19 kg) but not with placebo (+0.15 kg). However, there was no observed improvement in blood pressure in either group.

In conclusion, this study demonstrated that exenatide QWS-AI reduced HbA1c more than sitagliptin or placebo and was well-tolerated [142,143,144]. The main outcomes and side effects observed in clinical trials of GLP1-RA against obesity are summarized in Table 5.

## 8. Conclusions and Future Prospects

A better understanding of metabolic homeostasis in both healthy individuals and altered metabolic phenotype in T2DM will likely lead to the development of better treatments for T2DM. The role of the nervous system, genetics, and hormones involved in metabolic homeostasis (such as insulin, glucagon, GLP-1, and GIP), as well as glucotoxicity diets and feeding behaviors, sedentary lifestyle, altered islet cell behavior, altered extra pancreatic behavior, and risk factors (such as psychological stress), are part of the etiology and pathogenesis of T2DM. Given that T2DM is a multifactorial disease involving several hormones, their receptors, and subsequent intracellular activity, future therapeutic research must consider how the action of all these hormones interact synergistically in T2DM to produce the altered metabolic phenotype and also how treatments such as therapies based on GLP-1R activation can influence this hormonal synergism to produce a metabolic phenotype similar to that of a healthy individual. GLP-1R agonists are an attractive target for generating more effective therapies for T2DM, given their reported beneficial effects on several organs in the body involved in the pathology of the disease.

There is still potential for further research to improve and optimize the use of GLP-1 in diabetes and non-diabetic obesity to reduce morbidity and mortality associated with these metabolic disorders and improve the quality of life. However, some potential additional effects beyond weight loss could hypothetically contribute to some clinical outcomes, but evidence from human studies is lacking. The decreased incretin effect is common in several conditions accompanied by insulin resistance and is particularly well-documented in T2DM.

In most studies, GLP-1 levels were not related to insulin concentration or measures of insulin resistance. In preclinical models, GLP-1 mainly demonstrates a stimulatory effect on the HPG axis. Therefore, pharmacological stimulation of the GLP-1R by GLP-1RA might be able to reverse gonadotropin suppression in various states of metabolic imbalance.

Due to the complexity of biological systems, the final effect of GLP-1 on the HPG axis is multifactorial and appears to integrate other synergistic and counterbalancing metabolic and endocrine factors. In addition, GLP-1 appears to have a direct anti-fibrotic and anti-inflammatory effect on peripheral reproductive tissues.

More evidence based on real-life studies with long-term follow-up of therapeutic effectiveness, tolerability, and adverse effects given either by the mode of administration (injectable/oral) are still necessary. Moreover, particular attention should be paid to the way in which prescribing doctors apply the recommendations of international guidelines in order to avoid as much as possible clinical inertia, to improve cardio–reno metabolic efficiency and safety in obese patients with or without diabetes.

In conclusion, clinical studies and the anatomical distribution of GLP-1R suggest that GLP-1 might play a vital role as a modulatory signal between metabolic and reproductive systems. Management of comorbidities increasingly common in T2DM patients, such as obesity and liver disease, needs to be better addressed. In this regard, ongoing studies will provide further information on whether the benefits of GLP-1 extend to these indications.

## Figures and Tables

**Table 1:** Summary of clinical trials that investigated weight loss using liraglutide.

Study	Dosage/Number of Patients/Duration	Main Outcomes, Weight Reduction	Side Effects	Ref.
LEAD-1	liraglutide 0.6, 1.2, or 1.8 mg/day with sulfonylurea versus placebo N = 1041, 26 weeks	−0.2 kg (baseline 83.0 kg) for 1.8 mg and +0.3 kg (baseline 80.0 kg) for 1.2 mg/day, better glycemic control	Hypoglycemia, nausea, vomiting, diarrhea	[66]
LEAD-2	liraglutide 0.6, 1.2, or 1.8 mg/day with metformin as a background treatment N = 1091, 26 weeks	Body weight decreased in all groups (1.8–2.8 kg), comparable glycemic control	Minor hypoglycemia, nausea	[67]
LEAD-3	liraglutide 1.2 mg/day [N = 251] or 1.8 mg/day [N = 247], versus glimepiride, 52 weeks	A_1c_ decreased by 0.51% with glimepiride versus 0.84% with liraglutide 1.2 mg and 1.14% with liraglutide 1.8 mg	Vomiting	[68]
LEAD-4	liraglutide 1.2 or 1.8 mg/day versus placebo, N = 533, 26 weeks	Dose-dependent weight loss of 1–2 kg with 1.2 and 1.8 mg liraglutide versus weight gain of about 0.6 kg in placebo	Minor hypoglycemia, early gastrointestinal adverse events	[69]
LEAD-5	liraglutide 1.8 mg/day [N = 232], placebo [N = 115], and open-label insulin glargine [N = 234], all in combination with metformin (1 g/twice per day) and glimepiride (4 mg/day), 26 weeks	Average reduction of 1.8 kg in the liraglutide group versus 0.42 kg in the placebo group and a 1.6 kg increase in the glargine group.	Nausea (14%) reported in the liraglutide group	[67]
LEAD-6	liraglutide 1.8 mg/day [N = 233] or exenatide 10 μg twice/day [N = 231] open-label, parallel-group, multinational study (15 countries), 26 weeks	Average weight loss 3.24 kg (liraglutide group) and 2.87 kg (exenatide group)	Minor hypoglycaemia, less frequent in the liraglutide group compared to the exenatide one	[70]

**Table 2:** Summary of clinical trials that investigated weight loss using semaglutide.

Study	Dosage/Number of Patients/Duration	Main Outcomes, Weight Reduction	Side Effects	Ref.
STEP-1	Semaglutide 2.4 mg/week versus placebo N = 1961, 68 weeks	14.9% reduction of body weight versus 2.4% in placebo	Nausea and diarrhea	[102]
STEP-2	Semaglutide 2.4 mg, or 1.0 mg once/week versus placebo N = 1210, 68 weeks	9.64%, 6.99%, and 3.42% average body weight reduction with semaglutide 2.4 mg, 1.0 mg, and placebo, respectively.	Mild to moderate gastrointestinal adverse events more frequent with semaglutide 2.4 mg than with placebo	[103,115]
STEP 3	Semaglutide 2.4 mg once/week N = 611, 68 weeks	16.0% average weight reduction versus 5.7% with placebo. The co-primary endpoint of at least a 5% reduction in body weight was met by 86.6% versus 47.6%.	Gastrointestinal adverse events more frequent with semaglutide vs. placebo	[104]
STEP-4	Semaglutide 2.4 mg/week for the first 20 weeks, followed by random semaglutide or placebo for the remaining 48 weeks. N = 902, 68 weeks	A total weight loss of 5.0%	Gastrointestinal events in 49.1% of participants in semaglutide group vs. 26.1% in placebo	[43]
STEP-5	Semaglutide 2.4 mg versus placebo N = 304, 104 weeks	Decreasing weight until week 60, maintained through week 104; average placebo-corrected weight loss of 12.6 %	Gastrointestinal disorders, nausea, diarrhea, vomiting, and constipation more frequent in semaglutide group	[105]
STEP-6	Semaglutide 2.4 or 1.7 mg/week versus placebo in Asian people N = 401, 20 weeks	Body weight reduction 13.2%, 9.6%and 2.1%, respectively, for 2.4 mg, 1.7 mg, and placebo	Mild to moderate gastrointestinal disorders predominantly in semaglutide 2.4 group	[106,108]
STEP-7	Semaglutide 2.4 mg or placebo N = 375, 44 weeks	Not yet published	Not yet published	
STEP-8	Semaglutide 2.4 mg vs. liraglutide1.8 mg/day. N = 338, 68 weeks	Significantly greater bodyweight reduction; 15.8% with semaglutide, compared to 6.4% with liraglutide	Gastrointestinal adverse events in 84.1% participants in semaglutide group and 82.7% in liraglutide	[107]

**Table 3:** Summary of clinical trials that investigated weight loss using tirzepatide.

Study	Dosage/Number of Patients/Duration	Main Outcomes, Weight Reduction	Side Effects	Ref.
SURPASS-1	Tirzepatide 5, 10, and 15 mg/week. N = 705, 40 weeks	Dose-dependent bodyweight loss ranging from 7 to 9.5 kg	Transient gastrointestinal events, nausea, diarrhea, vomiting	[117]
SURPASS-2	Tirzepatide weekly doses (5, 10, and 15 mg) versus weekly injections of semaglutide 1.0 mg. N = 1879, 40 weeks	Greater reduction in body weight with tirzepatide than with semaglutide (−1.9 kg, −3.6 kg, and −5.5 kg, respectively)	Gastrointestinal events mild to moderate in both tirzepatide and semaglutide groups (nausea, diarrhea, vomiting)	[103]
SURPASS-3	Tirzepatide weekly (5, 10, and 15 mg) with daily insulin degludec in people with poorly controlled blood glucose despite stable treatment with metformin, with or without SGLT2 N = 1444, 52 weeks	Average loss of 7.5, 10.7, and 12.9 kg versus average weight gain of 2.3 kg in the degludec group	Mild to moderate gastrointestinal events	[119]
SURPASS-4	Tirzepatide 5 mg, 10 mg, or 15 mg/week or glargine 100 U/mL to reach fasting blood glucose < 100 mg/dLs N = 3045, 48 weeks	74–88% of people taking tirzepatide achieved HbA1c below 7.0% without weight gain or severe hypoglycemia, versus13% in glargine group	Nausea, diarrhea, decreased appetite, and vomiting more frequent with tirzepatide than with glargine	[120]
SURPASS-5	Tirzepatide 5, 10, or 15 mg/week in people taking insulin glargine for T2DM, with or without metformin. N = 475, 40 weeks	Average reduced body weight by 6.2, 8.2, and 10.9 kg, respectively	Gastrointestinal events, decreased appetite in 7–14% participants in tirzepatide group compared to 1.7% in placebo group, potentially contributing to weight loss	[121]
SURPASS-J-mono	Tirzepatide 5, 10, or 15 mg/week versus dulaglutide 0.75 mg/week in Japanese people with type 2 diabetes taking no other glucose-lowering medications during the study. N = 821, 96 weeks	Dose-dependent reduction of body weight in tirzepatide group (5.8 kg, 8.5 kg, and 10.7 kg, respectively), versus 0.5 kg reduction in dulaglutide group	Gastrointestinal events	[122]
SURPASS J-combo	Tirzepatide (5, 10, or 15 mg/week) in addition to non-incretin-based antidiabetic medications N = 484, 52 weeks	Dose-dependent reduction in body weight with tirzepatide compared with dulaglutide	Nausea, constipation, and nasopharyngitis	[123]
SURMOUNT-1	Tirzepatide 5, 10, or 15 mg/week in obese people without diabetes N = 2539, 72 weeks	15.0%, 19.5%, and 20.9%, respectively, body weight reduction in tirzepatide group, compared with just 3.1% in placebo	common gastrointestinal adverse events with tirzepatide	[124]

**Table 4:** Summary of clinical trials that investigated weight loss using Lixisenatide.

Study	Dosage/Number of Patients/Duration	Main Outcomes, Weight Reduction	Side Effects	Ref.
Get-Goal Mono	Lixisenatide 20 μg/day in medication-naïve people N = 361, 12 weeks	2 kg reduction regardless of treatment allocation.	Nausea	[130]
Get-Goal-M	Lixisenatide 20 μg once daily, as add-on therapy in patients with T2DM insufficiently controlled with metformin alone. N = 680, 24 weeks	Mean body weight decreased to a similar extent in all groups.	Nausea and vomiting more frequently in Lixisenatide group	[131]
Get-Goal-X	Lixisenatide 20 µg daily versus exenatide 10µg twice daily in T2DM inadequately controlled with metformin N = 1243, 24 weeks	25.1% of Lixisenatide patients and 31.4% of exenatide patients had ≥5% weight loss from baseline to week 24	Gastrointestinal symptoms, treatment discontinuation for 6.3% in the Lixisenatide group and 7.6% in exenatide group	[132]
Get Goal F1	(1) Lixisenatide one-step dose increase (10 μg once daily for two weeks, then 20 μg once daily; N= 161); (2) Lixisenatide two-step dose increase (10 μg once daily for one week, 15 μg once daily for one week, then 20 μg once daily; N = 161); (3) matching placebo one-step dose increase (N = 82); (4) matching placebo two-step dose increase (N = 80). N = 484, 24 weeks	Weight reduction between 2 kg and –2.7 kg in Lixisenatide group vs. 1.6 kg in placebo	Nausea and vomiting reported most frequently	[133]
Get Goal -S	Lixisenatide 20µg/day versus placebo inT2DM patients inadequately controlled with sulfonylurea ± metformin N = 1438, 24 weeks	≥5% weight loss from baseline to week 24 for 14.4% in Lixisenatide patients and 7.2% in placebo patients. Significant reduction in HbA_1c_ at week 24 versus placebo in Lixisenatide group	Nausea in Lixisenatide group, mainly in the first month of treatment	[134]
Get-Goal-P	Prandial Lixisenatide 20 µg/day versus placebo in T2DM patients insufficiently controlled by pioglitazone ± metformin. N = 484, 24 weeks	Average 0.2 kg reduction in body weight versus 0.2 kg increase in placebo group	Gastrointestinal disorders in Lixisenatide group	[135]
Get-Goal-L	Adding Lixisenatide (20µg/day) to established basal insulin therapy alone or together with metformin in people with T2DM and elevated glycated hemoglobin (HbA_1c_). N = 495, 24 weeks	Body weight decreased by 1.8 kg with Lixisenatide and 0.5 kg with placebo between randomization and week 24	Hypoglycemia and nausea were increased compared with placebo, but no excess of serious adverse events	[136]
Get-Goal Duo 1	Lixisenatide (20 µg/day) in patients with HbA_1c_ still elevated after initiation of insulin glargine	Statistically significant bodyweight increase by an average of 0.3 and 1.2 kg in Lixisenatide and placebo groups, respectively	Increase in the frequency of gastrointestinal side effects and modestly increased rates of hypoglycemia	[137]

**Table 5:** Summary of clinical trials that investigated weight loss using Exenatide.

Study	Dosage/Patients/Duration	Main Outcomes, Weight Reduction	Side Effects	Ref.
Duration-1	Exenatide 2 mg/week, against the pre-existing 10 µg/twice per day version N = 295, 30 weeks	No increased risk of hypoglycaemia and similar reductions in body weight	Nausea reported in both treatments, but more often for 10 µg/twice per day formulation	[136]
Duration-2	Exenatide (2 mg once/week) versus maximum approved doses sitagliptin, thiazolidinedione, or pioglitazone, in patients treated with metformin	Average 2.3 kg weight loss in exenatide group, 0.8 kg reduction in sitagliptin group, and 2.8 kg weight gain with pioglitazone	Nausea and diarrhea in exenatide and sitagliptin groups	[137]
Duration-3	Exenatide (2 mg once/week) versus insulin glargine titrated to glucose targets N = 456, 84 weeks	Average 2.6 kg decrease in bodyweight with exenatide, compared with a 1.4 kg increase with glargine, accompanied by improved glycemic control	No evidence	[145]
Duration-4	Exenatide once weekly (EQW) compared with metformin, pioglitazone, and sitagliptin (SITA) N = 820, 26 weeks	2.0 kg decrease with exenatide versus 0.8 kg reduction with sitagliptin and 1.5 kg increase with pioglitazone	Exenatide once weekly induced nausea and diarrhea	[146]
Duration-5	Exenatide (2 mg once/week)) versus exenatide twice daily (5 µg during 4 weeks followed by 10 µg during 20 weeks) in order to improve glycemic control, body weight, and safety. N = 252, 24 weeks	Similar reductions in mean body weight from baseline to wk 24 observed in both groups (−2.3 ± 0.4 kg and −1.4 ± 0.4 kg)	In both groups, the majority of nausea was transient and mild to moderate in intensity, while the incidence decreased over time	[138]
Duration-6	Exenatide once weekly (2 mg) versus liraglutide (1.8 mg) once daily in patients with T2DM. N = 911, 26 weeks	Better body weight reductions in liraglutide group (average 2.68–3.57 kg)	Nausea predominantly in exenatide group; diarrhea and vomiting more frequently in the liraglutide group and with decreasing incidence over time in both groups	[144]
Duration-7	Exenatide 2 mg once weekly or placebo in patients with T2DM inadequately controlled despite titrated insulin glargine ± metformin. N = 461, 28 weeks	Body weight reduction average of 1.5 kg with exenatide versus placebo.	Gastrointestinal and injection-site adverse events more frequent with exenatide + IG than with placebo + IG	[140]
Duration Neo-1	Exenatide 2 mg once/week, self-injectable Miglyol suspension (QWS-AI) versus exenatide 10 µg twice daily (BID), N = 375, 28 weeks	Significant body weight was reduced in both groups	Gastrointestinal adverse events were reported in 22.7% of patients within exenatide QWS-AI group and 35.6% in exenatide BID group	[141,142]
Duration-Neo-2	Exenatide 2 mg once-weekly Miglyol suspension for autoinjection (QWS-AI) versus sitagliptin (100 mg once/day oraly) or placebo. N = 364, 28 weeks	Average 1.12 kg and, respectively, 1.19 kg decrease of bodyweight in exenatide and sitagliptin groups versus 0.15 kg increase in the placebo	Gastrointestinal events and injection-site reactions	[142]

## Data Availability

Not applicable.
